# Design, synthesis and in vitro evaluation of novel bivalent *S*-adenosylmethionine analogues

**DOI:** 10.1016/j.bmcl.2011.11.017

**Published:** 2012-01-01

**Authors:** Catherine Joce, Rebecca White, Peter G. Stockley, Stuart Warriner, W. Bruce Turnbull, Adam Nelson

**Affiliations:** a School of Chemistry, University of Leeds, Leeds LS2 9JT, UK; b Astbury Centre for Structural Molecular Biology, University of Leeds, Leeds LS2 9JT, UK

**Keywords:** MetJ, Methionine repressor, *S*-Adenosylmethionine analogues, Bivalent ligands

## Abstract

In optimal cases, bivalent ligands can bind with exceptionally high affinity to their protein targets. However, designing optimised linkers, that orient the two binding groups perfectly, is challenging, and yet crucial in both fragment-based ligand design and in the discovery of bisubstrate enzyme inhibitors. To further our understanding of linker design, a series of novel bivalent *S*-adenosylmethionine (SAM) analogues were designed with the aim of interacting with the MetJ dimer in a bivalent sense (1:1 ligand/MetJ dimer). A range of ligands was synthesised and analyzed for ability to promote binding of the *Escherichia coli* methionine repressor, MetJ, to its operator DNA. Binding of bivalent SAM analogues to the MetJ homodimer in the presence of operator DNA was evaluated by fluorescence anisotropy and the effect of linker length and structure was investigated. The most effective bivalent ligand identified had a flexible linker, and promoted the DNA–protein interaction at 21-times lower concentration than the corresponding monovalent control compound.

## Graphical abstract

**Figure:**
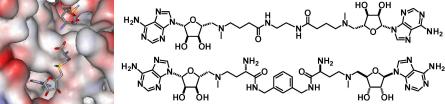

Bivalent ligands, in which two identical binding groups are linked by a spacer unit, can have exceptionally high binding affinity, and can, consequently, be useful modulators of biological function.^1^ However, the optimisation of linkers between the binding groups can be challenging, and, yet, is crucial in both fragment-based ligand (and drug) design^2^ and in the discovery of bisubstrate inhibitors.^3^ In this paper, we describe a series of symmetrical bivalent ligands that was prepared to extend our understanding of the effects of linker length and flexibility on biological activity.

*S*-Adenosylmethionine (SAM, **1**)^4,5^ was chosen as the binding unit due to its well studied interaction with the *Escherichia coli* methionine repressor, MetJ (Fig. 1). MetJ is a homodimeric DNA-binding protein which functions in complex with two molecules of the co-repressor, SAM. Binding of positively charged SAM molecules is believed to promote binding of the repressor to its target DNA by a unique mechanism based primarily on long-range electrostatics transferred through the protein structure.^6,7^ The dissociation constant of the MetJ-consensus, minimum operator DNA complex in the presence of saturating (1 mM) levels of SAM **1** has been determined to be 4 (±3) nM by filter binding studies. However, in the absence of SAM, **1**, saturation binding of the protein to the DNA was never achieved and the *K*_d_ was estimated to be 10 μM.^8^

The design of bivalent SAM analogues started by examination of the SAM–MetJ dimer–DNA complex crystal structure (Fig. 1).^4^ In this structure, a pair of co-repressor molecules is arranged symmetrically with the terminal carboxyl groups ∼5 Å apart. When designing the structure of bivalent SAM ligands, consideration was given to the structures of previously identified SAM analogues (Panel A, Fig. 2). Aza-SAM, **2**, is a stable nitrogen analogue of the (unstable^9^) natural co-repressor SAM, **1**, which has been shown to bind to the protein in an identical conformation.^10^ Previously, we have shown that both amide formation at the carboxy terminus of aza-SAM (to give **3**) and removal of the α-NH_2_ group (to give **4**) do not greatly affect function.^11^ In contrast, quaternisation of the 5′-position amine of **4**, to give the charged analogue **5**, significantly improved activity.^11^ A range of bivalent SAM derivatives was, therefore, designed in which secondary amides were used to link two aza-SAM analogues (Panel B, Fig. 2). The optimal separation of the terminal carbons in the linkers of such bivalent ligands may be estimated by analysis of the structure of the SAM–MetJ dimer–DNA complex. Provided that the bivalent ligands interact with MetJ analogously to SAM, and that the secondary amides adopt the preferred^12^ *syn* conformation, the optimal separation of the terminal carbons of the linker is ∼5.3 Å. The proposed interaction of an exemplar bivalent ligand to the MetJ dimer, in which a simple linker bridges between the binding units, is shown in Panel C, Figure 2. It was proposed to optimise the bivalent analogues by varying the length and flexibility of the linker and by investigating the effect of quaternisation.

Initial studies focused on bivalent analogues incorporating rigid linkers, for example linkers based on **7a** and **7b** (Scheme 1) in which the benzylic carbon atoms are separated by 5.8 and 5.0 Å respectively. Accordingly, coupling of the carboxylic acid **6** with the diamines **7a** and **7b** gave the bivalent derivatives **8a** and **8b** in 68% and 66% yield respectively; unfortunately, partial (∼20%) epimerization, α to the carbonyl group, occurred under the reaction conditions (Scheme 1).

The intermediates **8** were exploited in the synthesis of two different analogue classes. Acid-catalyzed deprotection gave the corresponding unquaternised ligands **9a**,**b**; alternatively, methylation and deprotection afforded the quaternised derivatives **10a**,**b** (Scheme 2). The bivalent ligands **9c**–**e** (Fig. 3 and Supplementary data) and the monovalent benzyl amides **11** and **12** were also prepared (Fig. 4 and Supplementary data).

It was also decided to prepare a series of bivalent ligands with more flexible linkers in which the length of the linker was varied more widely (see Scheme 3). The α-NH_2_ group was omitted for this series, both because this group has only a small^11^ effect on the function of monovalent amides (e.g., **3**) and because of the epimerization observed in couplings of α-NHBoc acids. Strain-promoted ‘click’ reaction^15^ between the azides **13** (*n* = 2^11^ and 4) and the cyclooctyne **14**^11^ and acetonide removal gave the corresponding bivalent analogues **15** as mixtures of regioisomers. Furthermore, reaction between the carboxylic acid^11^ **17** and a series of α,ω-diamines, and deprotection, gave the bivalent analogues **18** (*n* = 2, 3, 4 and 6). Finally, treatment of the bivalent analogues **15** and **18** with methyl iodide gave the corresponding quaternised derivatives **16** and **19**.

A fluorescence anisotropy-based binding assay was used to compare the relative ability of SAM analogues to promote MetJ dimer–DNA complex formation. Since SAM has low affinity for MetJ in the absence of DNA,^8,13^ measurements were made in the presence of the DNA fragment F-*metC*, a fluorescently-labeled analogue of the shortest naturally occurring operator sequence, *metC*.^14^ Titration of MetJ into a solution containing fixed concentrations of F-*metC* and ligand allows the change in anisotropy related to ternary complex formation to be measured (Fig. 5).

The half-maximal concentration of MetJ, EC_50_, required to promote complex formation was determined for each ligand. The concentration of the ligands used was comparable to the low millimolar SAM concentrations used in previous in vitro binding assays, and to the estimated SAM concentration in vivo.^8^ The monovalent ligands **11** and **12** (2 mM) and the bivalent ligands **9** and **10** (1 mM) all promoted the formation of the MetJ–DNA complex (Table 1). The EC_50_ was found to be 120 ± 10 nM and 36 ± 6 nM in the presence of the unquaternised and quaternised monovalent control ligands **11** and **12** respectively. The unquaternised bivalent ligands **9a**–**e** (1 mM) promoted complex formation up to about threefold more effectively than the corresponding monovalent analogue **11**. Similarly, the quaternised bivalent ligands **10a** and **10b** promoted, respectively, complex formation 2.4- and 3.6-fold more effectively than the quaternised monovalent analogue **12**. It is notable that the EC_50_ value of the ligand **10b** was significantly lower than that of the (unstable^9^) co-repressor, SAM. For these bivalent analogues with rigid linkers, the nature of the linker did not have a profound effect on function: in each case, the EC_50_ values were comparable to those of the corresponding monovalent controls (either **11** or **12**) and, consequently, it is unlikely that the linkers allow the binding groups to engage with the MetJ dimer in a bivalent sense.

The ability of the unquaternised ligands **4**, **15**, and **18** (2 mM) to promote the formation of the MetJ–DNA complex was also investigated using fluorescence anisotropy (Table 2). In the presence of the monovalent ligand **4**, the EC_50_ was 1000 ± 100 nM.^11^ Mixtures of the regioisomeric triazoles **15a** and **15b** promoted complex formation at 9- and 13-fold lower concentration than the monovalent ligand **4** respectively. In contrast, he activity of the bivalent ligands **18**—which had shorter and more flexible linkers than the triazoles **15**—depended critically on the length and nature of the linker; the compound with the shortest linker—**18a** in which *n* = 2—had similar activity to the monovalent derivative **4**. However, as the linker length increased, the EC_50_ improved from 700 ± 50 nM (with **18a**, *n* = 2) to 47 ± 1 nM (with **18d**, *n* = 6). Unfortunately, derivatives with longer linkers (*n* = 9 and 12) were not soluble under the conditions of the assay. The clear dependence of the activity of the ligands **18a**–**d** on the length of the linker is consistent with interaction with the MetJ dimer in a bivalent sense.

The ability of the quaternised ligands **16** and **19** (2 mM) to promote the formation of the MetJ–DNA complex was also investigated using fluorescence anisotropy (Table 3). The effect of the linker length was less profound with the quaternised analogues, generating only modest improvements in affinity of up to 3.5-fold. The EC_50_ improved from 150 ± 10 nM with the quaternised monovalent analogue **5** to 42 ± 3 nM with the most active quaternised bivalent derivative **16b**.

The results obtained in this study highlight the challenges associated with designing effective linkers in bivalent ligands. The activity of the bivalent ligands **9** and **10**, which have rather rigid linkers, was disappointing because the ligand concentrations required to promote DNA–protein interaction were comparable to those of the corresponding monovalent controls (**11** or **12**). For effective interaction, a rigid linker must control both the spacing and the relative orientation of the pair of binding groups: with **9** and **10**, it is likely that the linker design did not allow these ligands to engage with the MetJ dimer in a bivalent sense. However, in sharp contrast, the activity of the bivalent ligands, **18a**–**d** (*n* = 2, 3, 4, or 6) did depend critically on the length of the flexible linker. The effective length of flexible linkers in bivalent ligands has been estimated using alternative approaches including molecular dynamics simulations^16^ and random walk models.^17^ The optimal separation of the terminal carbons in the linkers of effective bivalent ligands **18** was estimated to be ∼5.3 Å (Fig. 1). However, the most effective unquaternised bivalent ligand **18** had *n* = 6 (i.e., **18d**), reinforcing that the effective lengths of flexible linkers are much shorter than their extended lengths.

Designing symmetrical ligands which interact effectively with their target in a bivalent sense is a difficult task, as observed previously,^18–20^ and highlighted again by this study. Here, modest affinity enhancements due to bivalency were only observed when flexible linkers were exploited: in the case of **18d**, a 21-fold increase in activity was observed compared to its monovalent analogue, **4**. We, therefore, find that simple, flexible linkers are a useful starting point in the design of bivalent ligands; once evidence of bivalent interaction with a target has been obtained with such ligands, optimisation of the linker may subsequently be possible. The development of strategies for designing effective linkers remains an important challenge, however, because of the large affinity enhancements that are possible with bivalent ligands in optimal cases.

## Figures and Tables

**Figure 1:**
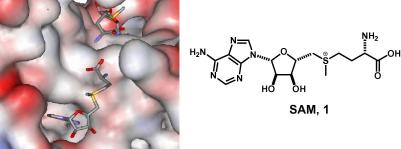
Co-crystal structure of two molecules of co-repressor SAM, **1**, bound on the surface of the MetJ dimer and the chemical structure of SAM.

**Figure 2:**
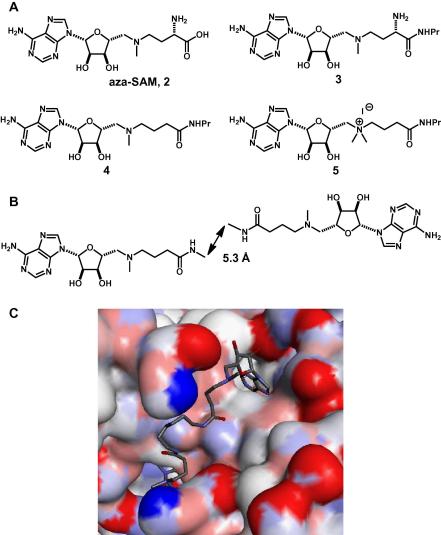
Design of bivalent ligands. (A) Monovalent ligands for the MetJ dimer. (B) Design of symmetrical bivalent SAM analogues; the optimal separation of the terminal carbon atoms in the linker was expected to be ∼5.3 Å. (C) Illustration of the possible interaction between an exemplar bivalent ligand and the MetJ homodimer. In this example, a simple linker joins the terminal carboxyls of two binding units analogues via secondary amides.

**Figure 3:**
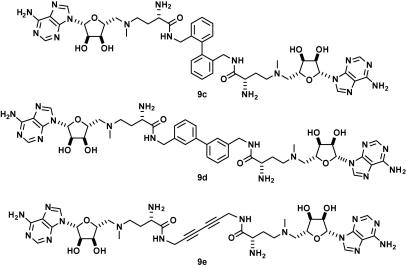
Structures of the bivalent ligands **9c**–**e**; partial epimerization occurred during the amide formation and the subsequent derivatives were isolated as mixtures of diastereoisomers.

**Figure 4:**
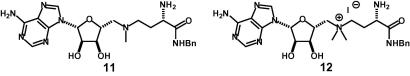
Structures of the monovalent ligands **11** and **12**; partial epimerization occurred during the amide formation and the subsequent derivatives were isolated as mixtures of diastereoisomers

**Figure 5:**
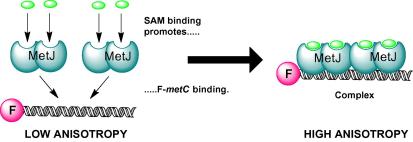
Cartoon illustrating the fluorescence anisotropy assay. The SAM molecules promote the formation of a SAM–F-*metC*–protein complex, with two MetJ dimers bound to the 18 base-pair DNA duplex.

**Scheme 1:**
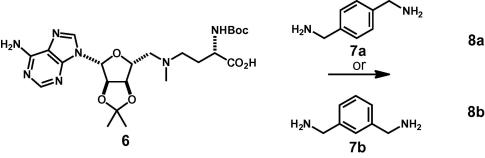
The intermediates **8a** and **8b** were prepared by coupling the carboxylic acid **6** and the diamines **7** (PyBOP, *^i^*Pr_2_NEt, DMF); partial (ca. 20%) epimerization was observed and all bivalent derivatives were consequently isolated as mixtures of diastereoisomers.

**Scheme 2:**
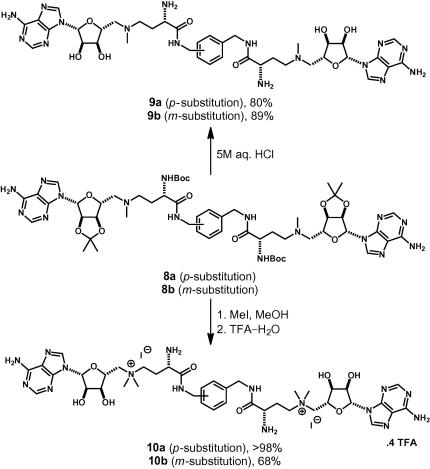
Synthesis of the bivalent ligands **9a**,**b** and **10a**,**b**.

**Scheme 3:**
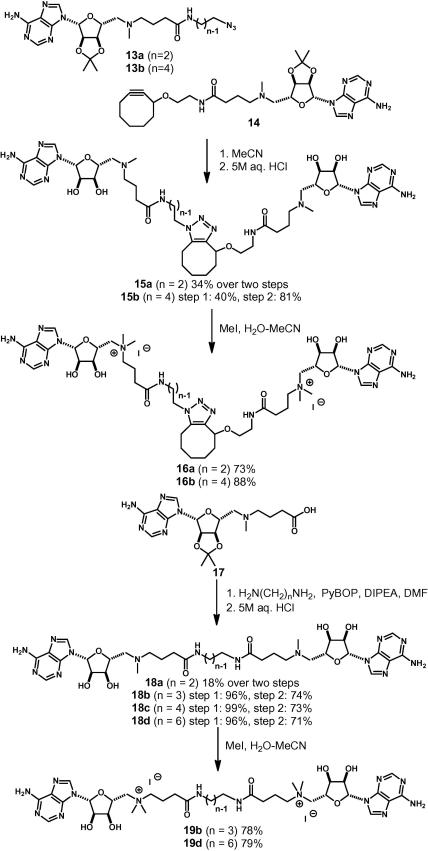
Synthesis of bivalent ligands; the triazoles **15** and **16** were prepared as mixtures of regioisomers.

**Table 1:** EC_50_ values, that is the concentration of MetJ monomer required to promote half-maximal formation of its complex with the F-*metC* DNA, in the presence of ligands (see Scheme 2 and Fig. 3); improvements in the activity of bivalent ligands relative to a monovalent control are shown

Compound	EC_50_ (nM)	Fold-improvement over monovalent control (**11** or **12**)
SAM, **1**	17.3 ± 0.3	—

**11**	120 ± 10	—
**9a**	61 ± 3	2.0-fold
**9b**	37 ± 2	3.2-fold
**9c**	39 ± 1	3.1-fold
**9d**	75 ± 4	1.6-fold
**9e**	111 ± 5	1.1-fold

**12**	36 ± 6	—
**10a**	15 ± 5	2.4-fold
**10b**	10 ± 1	3.6-fold

The concentration of the monovalent ligands **11** and **12** was 2 mM, and the concentration of the bivalent ligands **9** and **10** was 1 mM. The concentration of F-*metC* was 10 nM, and the final concentration of DMSO was 2%. EC_50_ values were determined based on an average of three titrations, fitted to a sigmoidal growth logistic model (see Supplementary data).

**Table 2:** EC_50_ values, that is the concentration of MetJ monomer required to promote half-maximal formation of its complex with the F-*metC* DNA, in the presence of ligands (see Scheme 3 and Fig. 2); improvements in the activity of bivalent ligands relative to a monovalent control are shown

Compound	EC_50_ (nM)	Fold-improvement over monovalent control **4**
**4**	1000 ± 100	—
**15a** (*n* = 2)	110 ± 6	9.1-fold
**15b** (*n* = 4)	76 ± 2	13-fold
**18a** (*n* = 2)	700 ± 50	1.4-fold
**18b** (*n* = 3)	320 ± 40	3.1-fold
**18c** (*n* = 4)	220 ± 20	4.5-fold
**18d** (*n* = 6)	47 ± 1	21-fold

The concentration of the ligands was 2 mM and the concentration of F-*metC* was 10 nM. EC_50_ values were determined based on an average of three titrations, fitted to a sigmoidal growth logistic model (see Supplementary data).

**Table 3:** EC_50_ values, that is the concentration of MetJ monomer required to promote half-maximal formation of its complex with the F-*metC* DNA, in the presence of ligands (see Scheme 3 and Fig. 2); improvements in the activity of bivalent ligands relative to a monovalent control are shown

Compound	EC_50_ (nM)	Fold-improvement over monovalent control **5**
**5**	150 ± 10	—
**16a** (*n* = 2)	50 ± 6	3.0-fold
**16b** (*n* = 4)	42 ± 3	3.6-fold
**19b** (*n* = 3)	108 ± 3	1.4-fold
**19d** (*n* = 6)	51 ± 2	2.9-fold

The concentration of the ligands was 2 mM and the concentration of F-*metC* was 10 nM. EC_50_ values were determined based on an average of three titrations, fitted to a sigmoidal growth logistic model (see Supplementary data).

## Supplementary data

Supplementary data Supplementary schemes, experimental details for the preparation of ligands and fluorescence anisotropy experiments.
