# Supplementary material for: Design, synthesis and in vitro evaluation of novel bivalent S-adenosylmethionine analogues
Source: Bioorg Med Chem Lett. 2012 Jan 1;22(1):278–84. doi: 10.1016/j.bmcl.2011.11.017 (PMC3267017; doi:10.1016/j.bmcl.2011.11.017)
Supplement: Supplementary data — Supplementary schemes, experimental details for the preparation of ligands and fluorescence anisotropy experiments. [file mmc1.doc]

Supplementary Material

Design, synthesis and *in vitro* evaluation of novel bivalent *S*-adenosylmethionine analogues

Catherine Joce, Rebecca White, Stuart Warriner, Peter G. Stockley, W. Bruce Turnbull and Adam Nelson

CONTENTS

Supplementary figures S2

General experimental S4

Experimental S6

Fluorescence anisotropy S35

Supplementary references S36

**Supplementary Figures:**

**Supplementary Figure 1:** Synthesis of monovalent benzyl amides **11** and **12**

**Supplementary Figure 2:** Synthesis of bivalent derivative **9c** with a rigid linker based on an *ortho*-substituted biaryl scaffold.

**Supplementary Figure 3:** Synthesis of bivalent derivative **9d** with a rigid linker based on a *meta*-substituted biaryl scaffold.

**Supplementary Figure 4:** Synthesis of bivalent derivative **9e** with a rigid linker based on a diyne scaffold.

**General Experimental**

THF was freshly distilled from sodium with benzophenone as an indicator. Dichloromethane and acetonitrile were distilled from CaH_2_. Ether refers to diethyl ether and petrol refers to petroleum spirit (b.p. 40-60 °C), unless otherwise stated. HPLC acetone, methanol and acetonitrile were used without distillation. Dry DMF and DMSO were obtained from a Sure-Seal bottle, stored under nitrogen. Saturated ammonia in methanol was prepared by bubbling ammonia gas through methanol for a minimum of 30 min. All other solvents and reagents were of analytical grade and used as supplied. Commercially available starting materials were obtained from Sigma–Aldrich, Fisher, Lancaster or Alfa Aesar. PyBOP was recyrstallised from CH_2_Cl_2_–ether.

All non-aqueous reactions were carried out using oven or flame dried glassware, under nitrogen unless otherwise stated. Solvents were removed *in vacuo* using a Büchi rotary evaporator attached to a Vacuubrand Vario CVC 2000 pump.

Flash column chromatography was carried out using silica (35-70 µm particles), according to the method of Still, Kahn and Mitra.^1^ Thin layer chromatography was carried out on commercially available pre-coated plates (Merck silica Kieselgel 60F_254_). Ion exchange chromatography was carried out using Supelco DSC-SCX resin. Preparative HPLC was performed using a Thermohypersil Hyperprep HS C18 column with a Gilson preparative HPLC system and a single wavelength detector.

Proton and carbon NMR spectra were recorded on a Bruker Advance DPX 300, Advance 500 or DRX500 spectrophotometer using an internal deuterium lock. Carbon NMR spectra were recorded with composite pulse decoupling using the waltz 16 pulse sequence. DEPT, COSY, HMQC and HMBC pulse sequences were routinely used to aid the assignment of spectra. Chemical shifts are quoted in parts per million downfield of tetramethylsilane, and coupling constants (*J*) are given in Hz. NMR spectra were recorded at 300 K unless otherwise stated.

Infra-red spectra were recorded using a Perkin Elmer spectrum one FT-IR spectrophotometer. Melting points were recorded on a Reichert hot stage microscope and are uncorrected. Microanalyses were carried out by staff in the School of Chemistry at the University of Leeds using a Carlo Erba 1108 automatic analyser. Optical activity measurements were recorded at room temperature on an AA‑1000 polarimeter; units for [α]20are 10^–1^ deg cm^2^ g^–1^ and are omitted. Mass spectra were recorded on a Micromass LCT-KA111 or Bruker MicrOTOF focus electrospray mass spectrometer. Isotopic distributions were as expected. Accurate molecular weights were generally obtained by staff in the School of Chemistry at the University of Leeds using electrospray mass spectrometry using reserpine as the lock mass and sodium iodide as the standard. All mass spectra quoted to four decimal places are high resolution spectra.

Analytical LC-MS was performed using a Waters X-Terra achiral column (MS C18, 5 µm, 50 × 4.6 mm) with a Waters 2525 pump, Waters 2996 photodiode array detector and a Waters Micromass ZQ mass spectrometer as the detector: (Method Ultraquick:1.2 mL/min; H_2_O:MeCN with 0.5% formic acid; 2.5 min, 95.5, 3.5 min, 5:95, 5 min, 8:2; Method G: 1.2 mL/min; H_2_O:MeCN with 0.5% formic acid; 1.5 min, 100:0, 6.0 min, 50:50, 6.4 min, 0:100, 9.0 min, 0:100, 10.0 min, 80:20; Method F: 1.2 mL/min; H_2_O:MeCN with 0.5% formic acid; 1.5 min, 100:0, 6.0 min, 30:70, 6.4 min, 0:100, 9.0 min, 0:100, 10.0 min, 80:20).

During the course of this work, a series of adenosine derivatives were synthesised. These have all been named according to the following numbering system:

During the course of this project, a series of bivalent ligands have been synthesised that contain inherent symmetry. Where two atoms are spectoscopically identical due to the symmetry of the molecule, for example the two 5′ carbons in the structure below, they will be reported as one peak.

**Experimental**

***N*,*N*′-(1,4-Phenylenebismethylene)bis{(2′*S*)-2′-*tert*-butoxycarbonylamino-4′-[5′′-deoxy-5′′-(methylamino)-2′′,3′′-*O*-(1′′′-methylethylidene)adenosyl]butanamide} 8a**

PyBOP (200 mg, 0.383 mmol) and DIPEA (67 µL, 0.38 mmol) were added to a stirred solution of *p*-xylylenediamine (**7a**) (18 mg, 0.13 mmol) and the acid **6**^2^ (200 mg, 0.383 mmol) in DMF (4 mL) and the reaction mixture was stirred at room temperature for 18 h. The crude product was purified by ion exchange chromatography (SCX), eluting with saturated ammonia in methanol solution, followed by flash chromatography (gradient elution: 3:97-6:94 saturated ammonia in methanol solution–CH_2_Cl_2_) to yield a mixture of the diamide and an unwanted side product. A second flash chromatography (gradient elution: 6:94-12:88 methanol–CH_2_Cl_2_) gave the *diamide* **8a** (100 mg, 68%; 4:1 mixture of diastereoisomers) as a colourless foam; *R*_f_ 0.17 (7:93 saturated ammonia in methanol solution–CH_2_Cl_2_); ν_max_/cm^−1^ (film) 3328, 3197, 2979, 2464, 1651, 1601 and 1417; [α]20+20.4 (*c* 1.00 in CH_2_Cl_2_); δ_H_ (500 MHz; methanol-*d*_4_) 8.29 (2H, s, Ade 8-H^maj^), 8.27 (2H, s, Ade 8-H^min^), 8.24 (2H, s, Ade 2-H), 7.24‑7.19 (4H, m, 2‑H, 3‑H, 5-H and 6-H), 6.18 (2H, d, *J*2.6, 1′′-H), 5.51-5.49 (2H, m, 2′′‑H^min^), 5.49‑5.45 (2H, m, 2′′-H^maj^), 5.03-5.01 (2H, m, 3′′-H^min^), 4.99 (2H, dd, *J*6.4 and 3.4, 3′′‑H^maj^), 4.43-4.31 (6H, m, 4′′‑H, 1-CH_2_ and 4-CH_2_), 4.13-4.08 (2H, m, 2′‑H), 2.68 (2H, dd, *J* 13.3 and 5.6, 5′′-H_A_), 2.63 (2H, dd, *J* 13.3 and 7.7, 5′′‑H_B_), 2.50‑2.41 (4H, m, 4′-H), 2.21 (6H, s, NMe), 1.94-1.85 (2H, m, 3′-H_A_), 1.76-1.68 (2H, m, 3′-H_B_), 1.60 (6H, s, CMe_2_), 1.44 (18H, s, *t*-Bu) and 1.39 (6H, s, CMe_2_); δ_C_ (75 MHz; methanol-*d*_4_) 175.0 (amide C=O), 158.0 (carbamate C=O), 157.7, 154.4 (Ade C-2), 150.6, 142.2 (Ade C-8), 139.2 (C-1 and C‑4), 129.0 (C-2, C-3, C-5 and C-6), 120.9, 116.0 (*C*Me_2_), 91.9 (C‑1′′^min^), 91.7 (C‑1′′^maj^), 86.3 (C‑4′′), 85.3 (C-2′′), 84.9 (C‑3′′), 81.0 (*C*Me_3_), 61.1 (C-5′′^min^), 60.9 (C‑5′′^maj^), 55.9 (C-4′), 55.2 (C-2′), 44.0 (1-CH_2_ and 4‑CH_2_), 43.2 (NMe^maj^), 43.0 (NMe^min^), 30.7 (C-3′), 29.1 (C*Me*_3_), 27.8 (C*Me*_2_) and 25.9 (C*Me*_2_); *m/z* (ES^+^) 1143.6 (100%, MH^+^), 572.3 (80%, MH_2_^2+^); (Found: MH^+^ 1143.6092; C_54_H_78_N_16_O_12_ requires MH^+^ 1143.6058). Analysis of the product by 500 MHz ^1^H NMR spectroscopy revealed that partial epimerisation occurred at the 2′-position under these conditions.

***N*,*N*′-(1,3-Phenylenebismethylene)bis{(2′*S*)-2′-*tert*-butoxycarbonylamino-4′-[5′′-deoxy-5′′-(methylamino)-2′′,3′′-*O*-(1′′′-methylethylidene)adenosyl]butanamide} 8b**

PyBOP (200 mg, 0.383 mmol) and DIPEA (67 µL, 0.38 mmol) were added to a stirred solution of *m*-xylylenediamine (**7b**) (17 µL, 0.13 mmol) and the acid **6**^2^ (200 mg, 0.383 mmol) in DMF (4 mL) and the reaction mixture was stirred at room temperature for 18 h. The crude product was purified by ion exchange chromatography (SCX), eluting with saturated ammonia in methanol solution, followed by flash chromatography (gradient elution: 4:96-6:94 saturated ammonia in methanol solution–CH_2_Cl_2_) to yield a mixture of the diamide and an unwanted side product. A second flash chromatography (gradient elution: 6:94-12:88 methanol–CH_2_Cl_2_) gave the *diamide* **8b** (96 mg, 66%; 4:1 mixture of diastereoisomers) as a colourless foam; *R*_f_ 0.26 (10:90 saturated ammonia in methanol solution–CH_2_Cl_2_); ν_max_/cm^−1^ (film) 3324, 3205, 2979, 2932, 2396, 1647 and 1475; [α]20+6.8 (*c* 1.00 in CH_2_Cl_2_); δ_H_ (500 MHz; methanol‑*d*_4_) 8.28 (2H, s, Ade 8-H), 8.24 (2H, s, Ade 2-H), 7.29-7.15 (4H, m, 2‑H, 4‑H, 5-H and 6-H), 6.18 (2H, br s, 1′′-H), 5.49-5.45 (2H, m, 2′′‑H), 5.00 (2H, dd, *J*6.0 and 3.4, 3′′-H), 4.43‑4.30 (6H, m, 4′′-H, 1-CH_2_ and 3-CH_2_), 4.16-4.08 (2H, m, 2′‑H), 2.75-2.66 (4H, m, 5′′-H), 2.54‑2.44 (4H, m, 4′-H), 2.24 (6H, s, NMe), 1.97-1.87 (2H, m, 3′-H_A_), 1.78-1.69 (2H, m, 3′‑H_B_), 1.60 (6H, s, CMe_2_), 1.44 (18H, s, *t*‑Bu) and 1.39 (6H, s, CMe_2_); δ_C_ (75 MHz; methanol‑*d*_4_) 175.0 (amide C=O_min_), 174.9 (amide C=O_maj_), 158.0 (carbamate C=O), 157.7, 154.4 (Ade C-2), 150.6, 142.2 (Ade C-8), 140.5 (C-1 and C-3), 130.1 (C-4 and C-6), 128.0 (C-2 or C-5), 127.6 (C-2 or C-5), 121.0, 116.0 (*C*Me_2_), 91.9 (C-1′′^min^), 91.8 (C‑1′′^maj^), 86.1 (C‑4′′), 85.4 (C-2′′), 84.9 (C-3′′), 81.0 (*C*Me_3_), 61.0 (C-5′′^min^), 60.8 (C-5′′^maj^), 55.9 (C-4′), 55.2 (C-2′), 44.3 (1‑CH_2_ and 3‑CH_2_), 43.2 (NMe^maj^), 43.0 (NMe^min^), 30.6 (C-3′), 29.1 (C*Me*_3_), 27.8 (C*Me*_2_) and 26.0 (C*Me*_2_); *m/z* (ES^+^) 1143.6 (10%, MH^+^) and 572.3 (100%, MH_2_^2+^); (Found: MH^+^ 1143.6006; C_54_H_78_N_16_O_12_ requires MH^+^ 1143.6058). Analysis of the product by 500 MHz ^1^H NMR spectroscopy revealed that partial epimerisation occurred at the 2′-position under these conditions.

***N*,*N*′-(1,4-Phenylenebismethylene)bis{(2′*S*)-2′-amino-4′-[5′′-deoxy-5′′-(methylamino)-adenosyl]butanamide} 9a**

The diamide **8a** (95 mg, 0.083 mmol; 4:1 mixture of diastereoisomers) was dissolved in 5M aqueous HCl solution (2 mL), and the reaction mixture was stirred at room temperature for 10 min. The reaction mixture was neutralised by the dropwise addition of 2M aqueous NaOH solution and the crude product was purified by ion exchange chromatography (SCX), eluting with saturated ammonia in methanol solution, to give the *diamide* **9a** (57 mg, 80%; 4:1 mixture of diastereoisomers) as a colourless foam; ν_max_/cm^−1^ (solid) 3195, 2939, 1635, 1599 and 1472; [α]20–22.8 (*c* 1.00 in DMSO); δ_H_ (500 MHz; DMSO‑*d*_6_) 8.38 (2H, t, *J* 5.6, amide NH), 8.34 (2H, s, Ade 8-H), 8.16 (2H, s, Ade 2-H), 7.28 (4H, br s, Ade NH_2_), 7.19-7.17 (4H, m, 2-H, 3-H, 5-H and 6-H), 5.87 (2H, d, *J* 5.5, 1′′-H), 5.52‑4.92 (4H, br s, 2′-NH_2_), 4.66‑4.63 (2H, m, 2′′-H), 4.23 (4H, d, *J* 5.6, 1-CH_2_ and 4-CH_2_), 4.12-4.08 (2H, m, 3′′-H), 4.02‑3.97 (2H, m, 4′′‑H), 3.28 (2H, dd, *J* 7.7 and 4.7, 2′-H), 2.72 (2H, dd, *J* 13.3 and 5.1, 5′′‑H_A_), 2.57‑2.51 (2H, m, 5′′‑H_B_), 2.46-2.41 (4H, m, 4′‑H), 2.19 (6H, s, NMe^min^), 2.16 (6H, s, NMe^maj^), 1.96-1.87 (2H, m, 3′‑H_A_^min^), 1.81-1.74 (2H, m, 3′-H_A_^maj^) and 1.56-1.48 (2H, m, 3′‑H_B_); δ_C_ (75 MHz; DMSO-*d*_6_) 175.0 (C=O^min^), 174.6 (C=O^maj^), 156.4, 153.0 (Ade C-2), 149.8, 140.2 (Ade C-8), 138.3 (C-1 and C-4), 127.5 (C-2, C‑3, C-5 and C-6), 119.5, 87.9 (C‑1′′), 82.7 (C‑4′′^min^), 82.7 (C-4′′^maj^), 73.0 (C-2′′), 72.3 (C-3′′), 60.0 (C-5′′), 55.0 (C-4′^min^), 54.9 (C-4′^maj^), 53.7 (C‑2′), 42.9 (NMe^maj^), 42.9 (NMe^min^), 42.1 (1‑CH_2_ and 4-CH_2_) and 32.2 (C‑3′); *m/z* (ES^+^) 885.4 (60%, MNa^+^), 863.4 (100%, MH^+^); (Found: MH^+^ 863.4368; C_38_H_54_N_16_O_8_ requires MH^+^ 863.4383).

***N*,*N*′-(1,3-Phenylenebismethylene)bis{(2′*S*)-2′-amino-4′-[5′′-deoxy-5′′-(methylamino)-adenosyl]butanamide} 9b**

The diamide **8b** (55 mg, 0.048 mmol; 4:1 mixture of diastereoisomers) was dissolved in 5M aqueous HCl solution (2 mL), and the reaction mixture was stirred at room temperature for 15 min. The reaction mixture was neutralised by the dropwise addition of 2M aqueous NaOH solution and the crude product was purified by ion exchange chromatography (SCX), eluting with saturated ammonia in methanol solution, to give the *diamide* **9b** (37 mg, 89%; 4:1 mixture of diastereoisomers) as a colourless foam; ν_max_/cm^−1^ (film) 3319, 3192, 2934, 1644, 1601 and 1476; [α]20–23.6 (*c* 1.00 in DMSO); δ_H_ (300 MHz; DMSO-*d*_6_) 8.62 (2H, br s, amide NH), 8.36 (2H, s, Ade 8‑H^min^), 8.35 (2H, s, Ade 8-H^maj^), 8.17 (2H, s, Ade 2-H), 7.30 (4H, br s, Ade NH_2_), 7.28-7.24 (1H, m, Ph), 7.18-7.11 (3H, m, Ph), 5.88 (2H, d, *J* 5.1, 1′′‑H), 4.68-4.64 (2H, m, 2′′‑H), 4.30‑4.24 (4H, m, 1‑CH_2_ and 3-CH_2_), 4.14-4.09 (2H, m, 3′′‑H), 4.05-4.01 (2H, m, 4′′-H), 4.52‑4.48 (2H, m, 2′-H), 2.74-2.58 (4H, m, 5′′-H), 2.53-2.45 (4H, m, 4′-H), 2.21 (6H, s, NMe^min^), 2.19 (6H, s, NMe^maj^), 1.98-1.90 (2H, m, 3′‑H_A_^min^), 1.87‑1.81 (2H, m, 3′-H_A_^maj^), 1.69‑1.62 (2H, m, 3′-H_B_^maj^) and 1.58‑1.51 (2H, m, 3′-H_B_^min^); δ_C_ (75 MHz; DMSO-*d*_6_) 172.6 (C=O), 156.4, 153.0 (Ade C-2), 149.7, 140.2 (Ade C‑8), 139.6 (C-1 and C-3), 128.6 (Ph CH), 126.6 (Ph CH), 126.1 (Ph CH), 119.6, 88.1 (C-1′′^maj^), 87.9 (C‑1′′^min^), 82.3 (C‑4′′), 73.0 (C-2′′^maj^), 72.9 (C-2′′^min^), 72.4 (C-3′′), 60.0 (C-5′′^maj^), 59.9 (C‑5′′^min^), 54.6 (C-4′), 53.4 (C-2′), 43.0 (NMe^min^), 42.7 (NMe^maj^), 42.4 (1‑CH_2_ and 3-CH_2_) and 30.7 (C‑3′); *m/z* (ES^+^) 432.2 (100%, MH_2_^2+^); (Found: MH_2_^2+^ 432.2245; C_38_H_54_N_16_O_8_ requires MH_2_^2+^ 432.2228).

***N*,*N*′-(1,4-Phenylenebismethylene)bis{(2′*S*)-2′-amino-4′-[5′′-deoxy-5′′-(dimethylamino)-adenosyl]butanamide}iodide tetratrifluoroacetic acid salt 10a**

Iodomethane (3.0 µL, 0.049 mmol) was added to a solution of the diamide **8a** (14 mg, 0.012 mmol; complex mixture of diastereoisomers) in methanol (0.4 mL) and the reaction mixture was stirred at room temperature for 8 h. Iodomethane (3.0 µL, 0.049 mmol) was added to the reaction mixture, and the solution was stirred at room temperature for 20 h. The reaction mixture was concentrated *in vacuo*, and the resulting residue was dissolved in water (0.5 mL) and trifluoroacetic acid (0.5 mL) and stirred at room temperature for 18 h. The reaction mixture was concentrated *in vacuo* to give the *salt* **10a** (20 mg, 100% over 2 steps; complex mixture of diastereoisomers) as a pale yellow glass; ν_max_/cm^−1^ (solid) 3262, 3080, 2459, 1665 and 1427; [α]20+14.0 (*c* 1.00 in water); δ_H_ (500 MHz; D_2_O) 8.50-8.43 (4H, m, Ade 2-H and Ade 8-H), 7.36-7.29 (4H, m, 2-H, 3-H, 5-H and 6-H), 6.19-6.03 (2H, m, 1′′-H), 4.92-4.72 (2H, m, 2′′-H), 4.66-4.32 (8H, m, 3′′-H, 4′′-H, 1‑CH_2_ and 4‑CH_2_), 4.19-3.68 (6H, m, 5′′-H and 2′-H), 3.61-3.54 (4H, m, 4′-H), 3.27 (6H, s, NMe_2_), 3.23 (6H, s, NMe_2_) and 2.51‑2.37 (4H, m, 3′-H); δ_C_ (75 MHz; D_2_O) 168.1 (amide C=O^maj^), 167.8 (amide C=O^min^), 163.3 (q, ^2^*J*_CF_ 39.8, trifluoroacetic acid C=O), 150.5, 148.5, 145.1 (Ade C-2), 143.9 (Ade C‑8^min^), 143.5 (Ade C‑8^maj^), 137.2, 128.5 (Ar CH^min^), 128.2 (Ar CH^maj^), 119.7 (min), 118.8 (maj), 114.9 (trifluoroacetic acid CF_3_), 91.0 (C‑1′′), 77.1 (C-4′′), 73.4 (C-2′′^min^), 73.3 (C‑2′′^maj^), 72.0 (C‑3′′^maj^), 71.9 (C-3′′^min^), 67.4 (C-5′′), 60.9 (C-4′^maj^), 60.8 (C‑4′^min^), 52.8 (NMe_2_^maj^), 52.6 (NMe_2_^min^), 52.1 (NMe_2_), 51.0 (C-2′), 43.5 (1‑CH_2_^maj^ and 4‑CH_2_^maj^), 43.3 (1‑CH_2_^min^ and 4‑CH_2_^min^), 24.9 (C-3′^maj^) and 24.8 (C-3′^min^); *m/z* (ES^+^) 446.3 (100%, M^2+^); (Found: M^2+^ 446.2404; C_40_H_60_N_16_O_8_ requires M^2+^ 446.2385).

***N*,*N*′-(1,3-Phenylenebismethylene)bis{(2′*S*)-2′-amino-4′-[5′′-deoxy-5′′-(dimethylamino)-adenosyl]butanamide}iodide tetratrifluoroacetic acid salt 10b**

Iodomethane (4.8 µL, 0.076 mmol) was added to a solution of the diamide **8b** (22 mg, 0.019 mmol; complex mixture of diastereoisomers) in methanol (0.5 mL) and the reaction mixture was stirred at room temperature for 16 h. The reaction mixture was concentrated *in vacuo*, and the resulting residue was dissolved in water (0.5 mL) and trifluoroacetic acid (0.5 mL), and the reaction mixture was stirred at room temperature for 18 h. The reaction mixture was concentrated *in vacuo* to give the *salt* **10b** (21 mg, 68% over 2 steps; complex mixture of diastereoisomers) as a pale yellow foam; ν_max_/cm^−1^ (solid) 3267, 3082, 1669, 1508 and 1430; [α]20+7.6 (*c* 1.00 in water); δ_H_ (300 MHz; D_2_O) 8.43-8.29 (4H, m, Ade 2-H and Ade 8-H), 7.26 (1H, t, *J* 7.5, 5-H), 7.14‑7.10 (3H, m, 2-H, 4-H and 6-H), 6.06-6.03 (2H, m, 1′′-H), 4.61‑4.58 (2H, m, 2′′-H^maj^), 4.53-4.51 (2H, m, 2′′-H^min^), 4.46-4.42 (4H, m, 3′′-H and 4′′-H), 4.30-4.20 (4H, m, 1‑CH_2_ and 3‑CH_2_), 4.05 (2H, app t, *J* 6.6, 2′-H), 3.99‑3.95 (2H, m, 5′′-H_A_^min^), 3.94-3.87 (2H, m, 5′′‑H_A_^maj^), 3.82-3.73 (2H, m, 5′′-H_B_), 3.49-3.43 (2H, m, 4′-H_A_), 3.41-3.35 (2H, m, 4′-H_B_) and 3.17‑3.11 (12H, m, NMe_2_); δ_C_ (75 MHz; D_2_O) 169.7 (amide C=O), 164.7 (q, ^2^*J*_CF_ 35.6, trifluoroacetic acid C=O), 152.2, 150.3, 146.8 (Ade C-2), 145.1 (Ade C‑8), 139.8, 131.5 (CH Ph), 129.2 (CH Ph), 128.9 (CH Ph), 120.4, 116.6 (trifluoroacetic acid CF_3_), 92.5 (C‑1′′), 78.7 (C-4′′), 74.7 (C-2′′), 73.6 (C-3′′), 69.1 (C-5′′), 62.4 (C-4′), 54.2 (NMe_2_), 53.7 (NMe_2_), 52.6 (C-2′), 45.4 (1-CH_2_ and 3‑CH_2_) and 26.5 (C‑3′); *m/z* (ES^+^) 446.7 (100%, M^2+^); (Found: M^2+^ 446.7433; C_40_H_60_N_16_O_8_ requires M^2+^ 446.7424).

**5′-[{(3′′*S*)-4′′-Benzylamino-3′′-*tert*-butoxycarbonylamino-4′′-oxobutyl}methylamino]-5′-deoxy-2′,3′-*O*-(1′′′-methylethylidene)adenosine S1**

PyBOP (100 mg, 0.192 mmol) and DIPEA (33 µL, 0.19 mmol) were added to a stirred solution of benzylamine (18 µL, 0.16 mmol) and the acid **6**^2^ (100 mg, 0.192 mmol) in DMF (2 mL) and the reaction mixture was stirred at room temperature for 18 h. The crude product was purified by ion exchange chromatography (SCX), eluting with saturated ammonia in methanol solution, followed by flash chromatography (gradient elution: 3:97-5:95 methanol–CH_2_Cl_2_) to give the *amide* **S1** (56 mg, 58%; 4:1 mixture of diastereoisomers) as a colourless foam; *R*_f_ 0.44 (10:90 methanol–CH_2_Cl_2_); ν_max_/cm^−1^ (film) 3323, 3192, 2978, 2802, 1646, 1599 and 1367; [α]20+18.0 (*c* 1.00 in CH_2_Cl_2_); δ_H_ (300 MHz; methanol-*d*_4_) 8.29 (1H, s, 8-H^min^), 8.28 (1H, s, 8‑H^maj^), 8.25 (1H, s, 2-H), 7.36‑7.24 (5H, m, Ph), 6.18 (1H, d, *J* 2.4, 1′‑H), 5.50-5.45 (1H, m, 2′‑H), 5.03-4.99 (1H, m, 3′‑H), 4.47-4.42 (1H, m, 4′-H), 4.37-4.32 (2H, m, C*H*_2_Ph), 4.16-4.14 (1H, m, 3′′-H^min^), 4.12-4.09 (1H, m, 3′′-H^maj^), 2.71-2.61 (2H, m, 5′‑H), 2.51-2.39 (2H, m, 1′′-H), 2.22 (3H, s, NMe), 1.95-1.84 (1H, m, 2′′-H_A_), 1.78‑1.70 (1H, m, 2′′‑H_B_), 1.61 (3H, s, CMe_2_^maj^), 1.60 (3H, s, CMe_2_^min^), 1.45 (9H, s, *t*-Bu^min^), 1.43 (9H, s, *t*‑Bu^maj^), 1.40 (3H, s, CMe_2_^min^) and 1.39 (3H, s, CMe_2_^maj^); δ_C_ (75 MHz; methanol-*d*_4_) 175.1 (amide C=O), 158.2 (carbamate C=O), 157.7, 154.3 (C‑2), 150.6, 142.2 (C-8), 140.2, 129.8 (Ph CH), 128.8 (Ph CH), 128.5 (Ph CH), 121.0, 116.0 (*C*Me_2_^maj^), 115.9 (*C*Me_2_^min^), 91.9 (C‑1′^min^), 91.7 (C‑1′^maj^), 86.3 (C‑4′), 85.3 (C-2′), 84.9 (C-3′^maj^), 84.9 (C-3′^min^), 80.9 (*C*Me_3_), 61.1 (C-5′^min^), 60.9 (C‑5′^maj^), 55.9 (C-1′′^maj^), 55.8 (C-1′′^min^), 55.3 (C-3′′^min^), 55.1 (C-3′′^maj^), 44.3 (*C*H_2_Ph), 43.2 (NMe^maj^), 43.1 (NMe^min^), 30.6 (C‑2′′), 29.0 (C*Me*_3_), 27.8 (C*Me*_2_) and 25.9 (C*Me*_2_); *m/z* (ES^+^) 611.3 (100%, MH^+^); (Found: MH^+^ 611.3278; C_30_H_42_N_8_O_6_ requires MH^+^ 611.3300). Analysis of the product by 500 MHz ^1^H NMR spectroscopy revealed that partial epimerisation occurred at the 3′′-position under these conditions.

**5′-[{(3′′*S*)-4′′-Benzylamino-3′′-amino-4′′-oxobutyl}methylamino]-5′-deoxyadenosine 11**

The amide **S1** (24 mg, 0.040 mmol; 4:1 mixture of diastereoisomers) was dissolved in 5M aqueous HCl solution (1 mL), and the reaction mixture was stirred at room temperature for 15 min. The reaction mixture was neutralised by the dropwise addition of 2M aqueous NaOH solution and the crude product was purified by ion exchange chromatography (SCX), eluting with saturated ammonia in methanol solution, to give the *amide* **11** (17 mg, 94%; 4:1 mixture of diastereoisomers) as a colourless film; ν_max_/cm^−1^ (film) 3307, 3153, 2917, 2467, 1641, 1574 and 1421; [α]20–22.0 (*c* 1.00 in DMSO); δ_H_ (500 MHz; DMSO‑*d*_6_) 8.38-8.33 (2H, m, 8-H and amide NH), 8.17 (1H, s, 2-H), 7.34‑7.21 (7H, m, Ph and Ade-NH_2_), 5.88 (1H, d, *J* 5.1, 1′‑H), 5.85-5.39 (2H, br s, 3′′‑NH_2_), 4.67‑4.62 (1H, m, 2′-H), 4.28 (2H, d, *J* 4.3, C*H*_2_Ph), 4.13-4.10 (1H, m, 3′-H), 4.03‑3.98 (1H, m, 4′-H), 3.23 (1H, dd, *J*7.7 and 4.7, 3′′‑H^maj^), 3.19‑3.17 (1H, m, 3′′-H^min^), 2.71 (1H, dd, *J* 13.3 and 4.7, 5′-H_A_), 2.54-2.50 (1H, m, 5′-H_B_), 2.46‑2.41 (2H, m, 1′′‑H), 2.18 (3H, s, NMe), 1.82-1.75 (1H, m, 2′′‑H_A_) and 1.54-1.46 (1H, m, 2′′‑H_B_); δ_C_ (75 MHz; DMSO‑*d*_6_) 175.6 (C=O), 156.4, 153.0 (C‑2), 149.8, 140.2 (C-8), 140.0, 128.6 (Ph CH), 127.5 (Ph CH), 127.0 (Ph CH), 119.5, 87.9 (C-1′), 82.8 (C‑4′^maj^), 82.7 (C‑4′^min^), 73.1 (C‑2′), 72.3 (C-3′), 60.1 (C-5′), 55.1 (C-1′′), 53.9 (C-3′′^min^), 53.8 (C-3′′^maj^), 43.0 (NMe^maj^), 42.8 (NMe^min^), 42.3 (*C*H_2_Ph) and 32.9 (C‑2′′); *m/z* (ES^+^) 471.2 (100%, MH^+^); (Found: MH^+^ 471.2475; C_22_H_30_N_8_O_4_ requires MH^+^ 471.2463).

**5′-[{(3′′*S*)-4′′-Benzylamino-3′′-amino-4′′-oxobutyl}dimethylamino]-5′-deoxyadenosine iodide ditrifluoroacetic acid salt 12**

Iodomethane (12 µL, 0.19 mmol) was added to a solution of the amide **S1** (29 mg, 0.047 mmol; 3:2 mixture of diastereoisomers) in methanol (0.5 mL) and the reaction mixture was stirred at room temperature for 18 h. Iodomethane (12 µL, 0.19 mmol) was added to the reaction mixture, and the solution was stirred at room temperature for 6 h and then concentrated *in vacuo*. The resulting residue was dissolved in water (0.5 mL) and trifluoroacetic acid (0.5 mL) and the reaction mixture was stirred at room temperature for 22 h. The reaction mixture was concentrated *in vacuo* to give the *salt* **12** (39 mg, 98% over 2 steps; 3:2 mixture of diastereoisomers) as pale yellow foam; ν_max_/cm^−1^ (film) 3315, 2349, 1773, 1666 and 1428; [α]20+8.8 (*c* 1.00 in water); δ_H_ (500 MHz; D_2_O) 8.30-8.26 (2H, m, 2-H and 8‑H), 7.25-7.08 (5H, m, Ph), 6.00 (1H, d, *J* 2.5, 1′-H^maj^), 5.90 (1H, d, *J* 2.4, 1′-H^min^), 4.54 (1H, dd, *J* 7.5 and 4.8, 2′-H^maj^), 4.47 (1H, dd, *J* 7.9 and 5.4, 2′-H^min^), 4.42-4.34 (2H, m, 3′-H and 4′‑H), 4.28-4.05 (2H, m, C*H*_2_Ph), 3.98 (1H, dd, *J* 7.3 and 5.9, 3′′-H^maj^), 3.91 (1H, dd, *J* 6.8 and 6.4, 3′′-H^min^), 3.88-3.66 (2H, m, 5′-H), 3.41-3.22 (2H, m, 1′′-H), 3.09 (NMe_2_^min^), 3.08 (NMe_2_^maj^), 3.03 (NMe_2_) and 2.34‑2.22 (2H, m, 2′′-H); δ_C_ (75 MHz; D_2_O) 167.9 (C=O^maj^), 167.8 (C=O^min^), 163.2 (q, ^2^*J*_CF_ 35.6, trifluoroacetic acid C=O), 150.3, 148.4, 145.0 (C-2), 143.6 (C-8^min^), 143.5 (C-8^maj^), 137.7 (min), 137.6 (maj), 129.7 (Ph CH^min^), 129.4 (Ph CH^maj^), 128.4 (Ph CH^min^), 128.1 (Ph CH^maj^), 127.7 (Ph CH), 119.6 (min), 118.7 (maj), 114.8 (trifluoroacetic acid CF_3_), 90.9 (C‑1′), 77.1 (C‑4′), 73.4 (C-2′^min^), 73.2 (C-2′^maj^), 72.0 (C‑3′^maj^), 71.8 (C-3′^min^), 67.6 (C-5′^maj^), 66.6 (C‑5′^min^), 60.9 (C‑1′′^min^), 60.6 (C-1′′^maj^), 52.7 (NMe_2_^maj^), 52.5 (NMe_2_^min^), 51.8 (NMe_2_), 51.0 (C‑3′′^min^), 50.9 (C‑3′′^maj^), 43.9 (*C*H_2_Ph^maj^), 43.6 (*C*H_2_Ph^min^), 24.9 (C-2′′^maj^) and 24.8 (C-2′′^min^); *m/z* (ES^+^) 485.2 (100%, M^+^); (Found: M^+^ 485.2599; C_23_H_33_N_8_O_4_ requires M^+^ 485.2619).

**5′-Deoxy-5′-*N*-methyl-*N*′-(4-[(4-hydroxybutyl)amino]butyramide)-2′,3′-*O,O*-(1-methylethylidene)adenosine S12**

4-Amino-1-butanol (377 µL, 4.06 mmol) was added to a solution of 5′-deoxy-5′-*N*-methyl-*N*′-(4-butyric acid)-2′,3′-*O,O*-(1-methylethylidene)adenosine^2^ (165 mg, 0.406 mmol) in DMF (1.5 mL), stirred 10 min, followed by addition of PyBOP (634 mg, 1.22 mmol) and DIPEA (213 µL, 1.22 mmol). The reaction was stirred for 20 min, the solvent removed *in vacuo* and the resulting crude solid purified by flash chromatography, eluting with 5% saturated ammonia in methanol solution, followed by ion exchange chromatography (SCX), eluting with saturated ammonia in methanol solution to afford the *alcohol* **S12** (117 mg, 60%) as a colourless glass; *R*_f_ 0.26 (10:90 saturated ammonia in methanol solution–CH_2_Cl_2_); ν_max_/cm^−1^ (film) 3324 (NH_2_ and OH), 3197 (NH_2_), 2938 (CH), 2867 (CH), 2807, 1650 (NH_2_), 1600 (C=O) and 1594; [α]20–2.4 (*c* 1.00 in methanol); δ_H_ (500 MHz; methanol-*d*_4_) 8.19 (1H, s, 8‑H), 8.13 (1H, s, 2-H), 6.07 (1H, d, *J* 2.1, 1′-H), 5.39 (1H, dd, 6.4 and 2.1, 2′-H), 4.88 (1H, dd, *J* 6.4 and 3.9, 3′-H), 4.27-4.20 (1H, m, 4′-H), 3.48-3.42 (2H, m, butyl 4-H_2_), 3.08-3.02 (2H, m, butyl 1-H_2_), 2.58-2.50 (2H, m, 5′-H_2_), 2.25 (2H, t, *J* 7.3, butyryl 4-H_2_), 2.11 (3H, s, NMe), 2.02 (2H, t, *J* 7.3, butyryl 2-H_2_), 1.64-1.52 (2H, m, butyryl 3-H_2_), 1.48 (3H, s, Me), 1.46-1.40 (4H, m, butyl 2-H_2_ and 3-H_2_) and 1.27 (3H, s, Me); δ_C_ (75 MHz; methanol-*d*_4_) 175.9 (butyryl C-1), 157.7 (C-6), 154.3 (C-2), 150.6 (C-4), 142.3 (C-8), 121.0 (C-5), 115.8 (*C*Me_2_), 91.9 (C-1′), 86.5 (C-4′), 85.3 (C-2′), 85.1 (C-3′), 63.0 (butyl C-4), 60.8 (C-5′), 58.6 (butyryl C-4), 43.1 (NMe), 40.5 (butyl C-1), 35.1 (butyryl C-2), 31.3 (butyl C-2 or C-3), 27.8 (Me), 27.2 (butyl C-2 or C-3), 25.9 (Me) and 24.4 (butyryl C-3); *m/z* (ES^+^) 478.3 (MH^+^); (Found: MH^+^ 478.2753; C_22_H_35_N_7_O_5_ requires MH^+^ 478.2772).

**5′-Deoxy-5′-*N*-methyl-*N*′-(4-[(4-azidobutyl)amino] butyramide)-2′,3′-*O,O*-(1-methylethylidene)adenosine 13b**

Methanesulfonic anhydride (62.0 mg, 0.356 mmol) was added to a solution of the alcohol **S18** (68 mg, 0.142 mmol) and triethylamine (59.0 µL, 0.426 mmol) in CH_2_Cl_2_ (1.5 mL) at 0 °C, stirred 30 min, methanesulfonic anhydride (62.0 mg, 0.356 mmol) was added, the reaction stirred for 15 min and the solvent removed *in vacuo*. The resulting crude mesylate was dissolved in DMSO (1.5 mL), sodium azide (277 mg, 4.26 mmol) added and the reaction mixture heated to 70 °C for 30 min. The cooled reaction mixture was diluted with water (5 ml) and the solution applied directly to an ion exchange column (SCX) eluting with saturated ammonia in methanol solution, followed by flash chromatography eluting with 10:90 saturated ammonia in methanol solution–CH_2_Cl_2_, followed by flash chromatography eluting with 5:95 saturated ammonia in methanol solution–CH_2_Cl_2_ to afford the *azide* **13b** (34 mg, 48%) as a colourless glass; *R*_f_ 0.54 (10:90 saturated ammonia in methanol solution–CH_2_Cl_2_); ν_max_/cm^−1^ (film) 3325 (NH_2_), 3181 (NH_2_), 2939 (CH), 2862 (CH), 2796, 2097 (N_3_), 1644 (NH_2_), 1599 (C=O) and 1574; [α]20–0.46 (*c* 3.50 in methanol); δ_H_ (500 MHz; methanol-*d*_4_) 8.18 (1H, s, 8-H), 8.13 (1H, s, 2-H), 6.07 (1H, d, *J* 2.6, 1′-H), 5.39 (1H, dd, *J* 6.4 and 2.6, 2′-H), 4.88 (1H, dd, *J*  6.4 and 3.4, 3′-H), 4.27-4.21 (1H, m, 4′-H), 3.22-3.19 (2H, m, butyl 4-H_2_), 3.06 (2H, t, *J* 6.8, butyl 1-H_2_), 2.56-2.53 (2H, m, 5′-H_2_), 2.26 (2H, t, *J* 7.7, butyramide 4-H_2_), 2.11 (3H, s, NMe), 2.02 (2H, t, *J* 7.3, butyramide 2-H_2_), 1.64-1.53 (2H, m, butyramide 3-H_2_), 1.51 (3H, s, Me), 1.51-1.40 (4H, m, butyl 2-H_2_ and 3-H_2_) and 1.28 (3H, s, Me); δ_C_ (75 MHz; methanol‑*d*_4_) 176.0 (butyramide C-1), 157.7 (C-6), 154.3 (C-2), 150.6 (C-4), 142.3 (C-8), 120.9 (C-5), 115.9 (*C*Me_2_), 91.9 (C-1′), 86.4 (C-4′), 85.3 (C-2′), 85.1 (C-3′), 60.8 (C-5′), 58.6 (butyramide C-4), 52.4 (butyl C-4), 43.1 (NMe), 40.0 (butyl C-1), 35.0 (butyramide C-2), 28.0 (butyl C-2 or C-3), 27.7 (Me), 27.6 (butyl C-2 or C-3), 25.8 (Me) and 24.3 (butyramide C-3); *m/z* (ES^+^) 503.3 (MH^+^); (Found: MH^+^503.2819; C_22_H_34_N_10_O_4_ requires MH^+^ 503.2837).

**4-(Adenosyl)[(methyl)amino)-*N*-(2-(4-(2-(4-(adenosyl)(methyl)amino)butanamido)ethoxy)-4,5,6,7,8,9-hexahydro-1*H*-cycloocta[*d*][1,2,3]triazol-1-yl)ethyl)butanamide and 4-(adenosyl)(methyl)amino)-*N*-(2-(9-(2-(adenosyl)(methyl)amino)butanamido)ethoxy)-4,5,6,7,8,9-hexahydro-1*H*-cycloocta[*d*][1,2,3]triazol-1-yl)ethyl)butanamide 15a**

A solution of 5′-deoxy-5′-*N*-methyl-*N*′-(4-[(2-azidoethyl)amino] butyramide)-2′,3′-*O,O*-(1-methylethylidene)adenosine (**13a**)^2^ (148 mg, 0.216 mmol) in acetonitrile (541 µL) was added to a flask charged with 5′-deoxy-5′-*N*-methyl-*N*′-{4-[2-(cyclooct-2-ynyloxy)ethanamine] butyramide}-2′,3′-*O,O*-(1-methylethylidene)adenosine (**14**)^2^ (148 mg, 0.216 mmol). The reaction was stirred for 16 h, diluted with CH_2_Cl_2_ (10 mL) and purified by flash chromatography (gradient elution: 8:92-10:90 saturated ammonia in methanol solution–CH_2_Cl_2_). The resulting triazoles were treated with 5M aqueous HCl solution (1 mL) for 10 min, neutralised by addition of 1M aqueous NaOH solution and the solution applied directly to an ion echange column (SCX), eluting with saturated ammonia in methanol solution to afford the *regioisomeric triazoles* **15a** (69 mg, 34%) as a colourless glass; *R*_f_ 0.10 (20:80 saturated ammonia in methanol solution–CH_2_Cl_2_); ν_max_/cm^−1^ (film) 3350 (NH_2_), 2940 (CH), 2862, 2807, 2510, 1647 (C=O) and 1576 (NH_2_); [α]20−2.6 (*c* 4.00 in methanol); δ_H_ (500 MHz; methanol-*d*_4_) 8.18 (2H, s, adenine 8-H), 8.17 (2H, s, adenine 8-H), 8.09 (4H, s, adenine 2-H), 5.91-8.88 (6H, m, adenosine 1′-H and CHO), 4.66-4.59 (8H, m, adenosine 2′-H and CH_2_), 4.42-4.31 (2H, m, ethoxy CH), 4.30-4.20 (2H, m, ethoxy CH), 4.17-4.12 (4H, m, adenosine 3′-H), 4.12-4.06 (4H, adenosine 4′-H), 3.58-3.31 (6H, m, CH and ethoxy CH), 3.30-3.20 (2H, m, CH), 3.22-3.19 (4H, m, CH), 2.94-2.85 (2H, m, CH), 2.74-2.68 (8H, m, adenosine 5′-H_2_), 2.68-2.60 (2H, m, CH), 2.41-2.31 (8H, m, butanamide 4-H_2_), 2.21 (3H, s, NMe), 2.19 (9H, s, NMe), 2.13-1.99 (8H, m, butanamide 2-H_2_), 1.92-1.84 (4H, m, CH), 1.70‑1.56 (8H, m, butanamide 3-H_2_), 1.55-1.35 (4H, m, CH) and 1.15-0.96 (4H, m, CH); δ_C_ (75 MHz; methanol-*d*_4_) 176.5 (C=O), 176.2 (C=O), 157.7 (adenine C-6), 154.4 (adenine C‑2), 151.0 (adenine C-4), 146.3 (triazole), 145.8 (triazole), 142.0 (adenine C-8), 136.5 (triazole), 135.7 (triazole), 121.1 (adenine C-5), 91.0 (adenosine C-1′), 83.8 (adenosine C-2′), 76.0 (adenosine C-3′), 75.1 (adenosine C-3′), 74.1 (adenosine C-4′), 73.1 (adenosine C-4′), 68.7, (CH_2_), 65.1 (adenosine C-5′), 58.8 (butanamide C-4), 50.3 (NMe), 43.5 (NMe), 41.1 (CH_2_), 40.6, (CH_2_), 36.8 (CH_2_), 35.2 (CH_2_), 32.2 (CH_2_), 29.7 (CH_2_), 28.4 (CH_2_), 27.1 (CH_2_), 25.8 (CH_2_), 25.4 (CH_2_), 24.3 (CH_2_), 24.2 (CH_2_), 22.8 (CH_2_), 21.7 (CH_2_) and 48 carbons missing or overlapped; *m/z* (ES^+^) 475.9 (100% MH_2_^2+^) and 950.6 (MH^+^); (Found: MH^+^  950.5071; C_42_H_63_N_17_O_9_ requires MH^+^  950.5067). The similarity of the compounds isoloated means that a significant number of NMR peaks are overlapped.

**4-((Adenosyl)(methyl)amino)-*N*-(2-(1-(4-(4-((adenosyl) (methyl)amino)butanamido)butyl)-4,5,6,7,8,9-hexahydro-1*H*-cycloocta[*d*][1,2,3]triazol-4-yloxy)ethyl)butanamide and 4-(-((adenosyl)(methyl)amino)-*N*-(2-(1-(4-(4-(-((adenosyl)(methyl)amino)butanamido)butyl)-4,5,6,7,8,9-hexahydro-1H-cycloocta[d][1,2,3]triazol-9-yloxy)ethyl)butanamide 15b**

5′-Deoxy-5′-*N*-methyl-*N*′-{4-[2-(cyclooct-2-ynyloxy)ethanamine] butyramide}-2′,3′-*O,O*-(1-methylethylidene)adenosine (**14**)^2^ (164 mg, 0.295 mmol) in acetonitrile (0.86 mL) was added to a flask charged with the azide **13b** (148 mg, 0.295 mmol). The reaction was stirred for 18 h, diluted with CH_2_Cl_2_ (3 mL) and purified by flash chromatography (gradient elution: 5:95-8:92 saturated ammonia in methanol solution–CH_2_Cl_2_) to afford the *intermediate regioisomeric triazoles* as a colourless glass (125 mg, 40%); *R*_f_ 0.15 (10:90 saturated ammonia in methanol solution–CH_2_Cl_2_); ν_max_/cm^−1^ (film) 3335 (NH_2_), 3203 (NH_2_), 2978 (CH), 2936 (CH), 2861, 1796, 1646 (C=O) and 1600 (NH_2_); [α]20−4.8 (*c* 4.00 in methanol); δ_H_ (500 MHz; methanol-*d*_4_) 8.19 (4H, s, adenine 8-H), 8.12 (4H, s, adenine 2-H), 6.07 (4H, app. s, adenosine 1′-H), 5.39 (4H, dd, *J* 6.4 and 2.1, adenosine 2′-H), 4.89-4.85 (4H, m, adenosine 3′-H), 4.70-4.62 (2H, m, CHO), 4.30-4.15 (8H, m, adenosine 4′-H and CH), 3.35-3.26 (8H, m, CH), 3.10-3.02 (4H, m, CH), 2.97-2.88 (2H, m, CH), 2.73-2.62 (2H, m, CH), 2.58-2.47 (8H, m, adenosine 5′-H), 2.29-2.21 (8H, m, butanamido 4-H_2_), 2.13-2.08 (12H, m, NMe), 2.08-1.98 (10H, butanamido 3-H_2_ and CH), 1.95-1.83 (2H, m, CH), 1.83-1.69 (4H, m, CH), 1.69-1.49 (12H, m, butanamido 2-H_2_ and CH), 1.47 (12H, s, Me), 1.45-1.30 (8H, m, CH), 1.27 (12H, s, Me) and 1.12-0.97 (4H, m, CH); δ_C_ (75 MHz; methanol-*d*_4_) 174.6 (butanamido C-1), 174.5 (butanamido C-1), 156.2 (adenine C-6), 152.8 (adenine C-2), 149.1 (adenine C-4), 144.8 (triazole), 144.4 (triazole), 140.8 (adenine C-8), 134.4 (triazole), 133.7 (triazole), 119.4 (adenine C-5), 114.3 (*CMe*_2_), 90.4 (adenosine C-1′), 84.9 (adenosine C-4′), 83.5 (adenosine C-3′), 74.4 (CHO), 67.1 (CH), 66.9 (CH), 59.3 (adenosine C-5′), 57.1 (butanamido C-4), 41.6 (NMe), 39.4 (CH), 39.3 (CH), 38.4 (CH), 38.3 (CH), 35.2 (CH), 33.5 (butanamido C-2), 28.3 (CH), 27.4 (CH), 27.0 (CH), 26.4 (CH), 26.3 (CH), 25.4 (Me), 24.4 (CH), 23.6 (Me), 22.8 (CH), 22.7 (CH), 21.2 (CH), 20.0 (CH) and 61 signals missing or overlapped; *m/z* (ES^+^) 529.8 (100% [M−2I^−^]^2+^) and 4058.6 [M−2I^−^]^+^; (Found: [M−2I^−^]^+^ 1058.5990; C_50_H_75_N_17_O_9_ requires [M−2I^−^]^+^ 1058.6006). The similarity of the compounds isoloated means that a significant number of NMR peaks are overlapped.

The *intermediate regioisomeric triazoles* (125 mg, 0.118 mmol) were treated with 5M aqueous HCl solution (1 mL) for 10 min, neutralised by addition of 1M aqueous NaOH solution and the solution applied directly to an ion exchange column (SCX), eluting with saturated ammonia in methanol solution to afford the *regioisomeric triazoles* **15b** (94 mg, 81%) as a colourless glass; *R*_f_ 0.48 (30:70 saturated ammonia in methanol solution–CH_2_Cl_2_); ν_max_/cm^−1^ (film) 3330 (NH_2_), 2937 (CH), 2863 (CH), 2807, 1647 (NH_2_) and 1603 (C=O); [α]+17.2 (*c* 2.00 in 1:1 water–methanol); δ_H_ (500 MHz; methanol-*d*_4_) 8.17 (4H, s, adenine 8-H), 8.10 (4H, s, adenine 2-H), 5.89 (4H, d, *J* 4.3, adenosine 1′-H), 4.68-4.60 (6H, m, adenosine 2′-H and CHO), 4.26 (2H, t, *J* 7.3, CH), 4.20-4.12 (6H, m, adenosine 3′-H and CH), 4.12-4.06 (4H, m, adenosine 4′-H), 3.54-3.26 (4H, m, CH), 3.07-3.00 (4H, m, CH), 2.95-2.86 (2H, m, CH), 2.75-2.68 (8H, m, adenosine 5′-H_2_), 2.68-2.60 (2H, m, CH), 2.41-2.33 (10H, m, butanamideo 4-H_2_ and CH), 2.22-2.17 (12H, m, NMe), 2.12-2.02 (8H, m, butanamido 2-H_2_), 1.93-1.57 (22H, butanamido 3-H_2_ and CH) and 1.56-1.29 (12 H, m, CH); δ_C_ (75 MHz; methanol-*d*_4_) 176.1 (butanamido C-1), 176.0 (butanamido (C-1), 157.6 (adenine C-6), 154.2 (adenine C-2), 150.9 (adenine C-4), 146.3 (triazole), 145.9 (triazole), 141.9 (adenine C-8), 135.9 (triazole), 135.3 (triazole), 121.0 (adenine C-5), 90.9 (adenosine C-1′), 83.6 (adenosine C-4′), 75.9 (adenosine C-2′), 75.0 (adenosine C-2′), 74.0 (adenosine C-3′), 72.9 (adenosine C-3′), 68.6 (CH), 68.4 (CH), 61.1 (adenosine C-5′), 61.0 (adenosine C-5′), 58.7 (butanamido C-4), 43.4 (NMe), 43.3 (NMe), 40.9, 40.7, 39.9, 39.8, 36.7, 35.1 (butanamido C-2), 35.0 (butanamido (C-2), 32.9, 29.8, 28.9, 28.5, 27.9, 27.8, 26.9, 25.9, 25.1, 24.2 (butanamido C-3), 23.8, 22.8, 21.5 and 44 signals missing or overlapped; *m/z* (ES^+^) 489.8 (100% MH_2_^2+^); (Found: MH^+^ 978.5408; C_44_H_67_N_17_O_9_ requires MH^+^ 978.5380). The similarity of the compounds isoloated means that a significant number of NMR peaks are overlapped.

**4-(Adenosyl)[(dimethyl)amino)-*N*-(2-(4-(2-(4-(adenosyl)(dimethyl)amino)butanamido)ethoxy)-4,5,6,7,8,9-hexahydro-1*H*-cycloocta[*d*][1,2,3]triazol-1-yl)ethyl)butanamide diiodide and 4-(adenosyl)(dimethyl)amino)-*N*-(2-(9-(2-(adenosyl)(dimethyl)amino)butanamido)ethoxy)-4,5,6,7,8,9-hexahydro-1*H*-cycloocta[*d*][1,2,3]triazol-1-yl)ethyl)butanamide diiodide 16a**

Methyl iodide (5.3 µL, 0.84 mmol) was added to a solution of the regioisomeric triazoles **15a** (40 mg, 0.42 mmol) in acetonitrile (0.5 mL) and water (0.2 mL). The reaction was stirred for 1 d, methyl iodide (2.7 µL, 0.42 mmol) added, stirred 1 d, methyl iodide (1.4 µL, 0.21 mmol) added, stirred 1 d, methyl iodide (2.7 µL, 0.42 mmol) added, stirred 1 d, methyl iodide (5.3 µL, 0.84 mmol) added, stirred 1 d, methyl iodide (4.05 µL, 0.63 mmol) added, stirred 1 d, methyl iodide (8.1 µL, 1.05 mmol) added, stirred 1 d and concentrated *in vacuo*. The reaction was redissolved in acetonitrile (0.3 mL) and water (0.2 mL), methyl iodide (1.4 µL, 0.21 mmol) was added and the reaction was stirred for 1 d. Methyl iodide (5.3 µL, 0.84 mmol) was added, the reaction was stirred for 3 d and concenctrated *in vacuo* to afford the *regioisomeric triazoles* **16a** as a colourless glass (38 mg, 73%); *R*_t_ (Method: Analysis G) 4.84 min; ν_max_/cm^−1^ (film) 3331 (NH_2_), 2923 (CH), 2862 (CH), 1634 (NH_2_) and 1596 (C=); [α]20+6.0 (*c* 3.00 in 1:1 water–methanol); δ_H_ (500 MHz; D_2_O) 8.25-8.21 (4H, m, adenine), 8.17-8.14 (4H, m, adenine), 6.12-6.04 (4H, m, adenosine 1′-H), 4.94-4.70 (10H, m), 4.63-4.53 (4H, m), 4.51-4.39 (4H, m), 4.38-4.33 (1H, m), 4.33-4.28 (2H, m), 4.08-3.86 (4H, m), 3.85-3.71 (4H, m), 3.64-3.31 (14H, m), 3.28-3.11 (26H, m, including NMe), 2.98-2.79 (3H, m), 2.74-2.61 (2H, m), 2.30-1.76 (19H, m), 1.71-1.61 (3H, m), 1.57-1.38 (7H, m), 1.33-1.22 (1H, m), 1.18-1.05 (1H, m) and 1.05-0.95 (1H, m); δ_C_ (75 MHz; D_2_O) 176.5 (butanamido C-1), 158.3 (adenine C-6), 155.6 (adenine C-2), 151.4 (adenine C-4), 148.1 (triazole), 147.3 (triazole), 143.1 (adenine C-8), 121.7 (adenine C-5), 92.3 (adenosine C-1′), 79.7 (adenosine), 76.8, 75.1 (adenosine), 74.7 (adenosine), 73.9, 69.4, 69.1, 68.7, 68.5, 68.3, 66.9, 55.1, (NMe), 54.9 (NMe), 54.4 (NMe), 50.8, 49.8, 42.0, 41.6, 36.7, 34.2, 29.1, 27.7, 26.4, 26.0, 24.6, 24.0, 23.0, 21.2, 21.1 and 50 peaks missing or overlapped; *m/z* (ES^+^) 489.8 (100% [M−2I^−^]^2+^); (Found: [M−2I^−^]^2+^) 489.7720; C_44_H_69_N_17_O_9_I_2_ requires [M−2I^−^]^2+^ 489.7727). The similarity of the compounds isoloated means that a significant number of NMR peaks are overlapped.

**4-((Adenosyl)(dimethyl)amino)-*N*-(2-(1-(4-(4-((adenosyl) (dimethyl)amino)butanamido)butyl)-4,5,6,7,8,9-hexahydro-1*H*-cycloocta[*d*][1,2,3]triazol-4-yloxy)ethyl)butanamide diiodide and 4-(-((adenosyl)(dimethyl)amino)-*N*-(2-(1-(4-(4-(-((adenosyl)(dimethyl)amino)butanamido)butyl)-4,5,6,7,8,9-hexahydro-1H-cycloocta[d][1,2,3]triazol-9-yloxy)ethyl)butanamide diiodide 16b**

Methyl iodide (7.6 µL, 0.12 mmol) was added to a solution of the regioisomeric triazoles **15b** (60 mg, 0.061 mmol) in acetonitrile (0.3 mL) and water (0.1 mL). The reaction was stirred for 1 d, methyl iodide (3.8 µL, 0.061 mmol) added, the reaction stirred 1 d and the solvent removed *in vacuo* to afford the *regioisomeric triazoles* **16b** (68 mg, 88%) as a colourless glass; *R*_t_ (Method: Analysis G) 5.14 min; ν_max_/cm^−1^ 3317 (NH_2_), 2936 (CH), 2862 (CH), 1645 (NH_2_) and 1596 (C=O); [α]20+3.6 (*c* 2.00 in 1:1 water–methanol); δ_H_ (500 MHz; D_2_O) 8.20-8.13 (4H, m, adenine 8-H), 8.08-7.98 (4H, m, adenine 2-H), 6.02-5.95 (4H, m, adenosine 1′‑H), 4.70-4.61 (4H, m, adenosine 2′-H), 4.56-4.47 (4H, m, adenosine 4′-H), 4.41-4.33 (4H, m, adenosine 3′-H), 4.23-4.10 (4H, m, CH), 4.00-3.90 (4H, m, adenosine 5′-H_A_), 3.70 (4H, app. d, *J* 14.1, adenosine 5′-H_B_), 3.50-3.25 (12H, m, CH), 3.14 (14H, s, NMe and CH), 3.11 (12H, s, NMe), 2.96-2.88 (4H, m, CH), 2.87-2.72 (2H, m, CH), 2.67-2.56 (2H, m, CH), 2.23-1.80 (14H, m, CH), 1.78-1.60 (8H, m, CH), 1.60-1.41 (6H, m, CH), 1.41-1.30 (5H, m, CH), 1.30-1.19 (5H, m, CH), 1.18-1.08 (2H, m, CH), 1.05-0.94 (2H, m, CH) and 0.90-0.77 (2H, m, CH); δ_C_ (75 MHz; D_2_O) 174.2 (butanamido C-1), 174.1 (butanamido C-1), 174.0 (butanamido C-1), 155.8 (adenine C-6), 153.2 (adenine C-2), 148.9 (adenine C-4), 145.9 (triazole), 145.8 (triazole), 140.7 (adenine C-8), 135.9 (triazole), 134.4 (triazole), 119.3 (adenine C-5), 89.8 (adenosine C-1′), 77.3 (adenosine C-4′), 74.6 (adenosine C-2′), 72.8 (adenosine C-2′), 72.3 (adenosine C-3′), 71.5 (adenosine C-3′), 67.1 (adenosine C-5′), 66.0 (adenosine C-5′), 64.6 (butanamido C-4), 52.6 (NMe), 52.2 (NMe), 48.8, 47.8, 39.7, 39.0, 34.4, 32.0, 28.1, 27.2, 27.1, 25.8, 25.3, 21.8, 20.7, 19.0 and 55 peaks missing or overlapped; *m/z* (ES^+^) 503.8 (100% [M−2I^−^]^2+^); (Found: [M−2I^−^]^2+^) 503.7867; C_46_H_73_N_17_O_9_I_2_ requires [M−2I^−^]^2+^ 503.7883). The similarity of the compounds isoloated means that a significant number of NMR peaks are overlapped.

***N*,*N*′-Di-[5′-deoxy-5′-*N*-methyl-*N*′-(4-butyryl)-adenosine]-1,2-ethylenediamine 18a**

Ethylene diamine (18 µL, 0.26 mmol) was added to a solution of 5′-deoxy-5′-*N*-methyl-*N*′-(4-butyric acid)-2′,3′-*O,O*-(1-methylethylidene)adenosine^2^ (322 mg, 0.794 mmol) in DMF (3 mL), stirred 10 min, followed by addition of PyBOP (412 mg, 0.792 mmol) and DIPEA (138µL, 0.792 mmol). The reaction was stirred for 17 h, the solvent removed *in vacuo* to yield a crude solid which was purified by ion exchange chromatography (SCX), eluting with saturated ammonia in methanol solution, followed by flash chromatography, eluting with 7.5:92.5 saturated ammonia in methanol solution–CH_2_Cl_2_, followed by HPLC (15-30% MeCN with 0.1% TFA in water with 0.1% TFA, Thermohypersil Hyperprep HS C18 column). Partial deprotection of the acetonide group occurred during HPLC so the resulting mixture was treated with 5M aqueous HCl solution (5 mL) for 15 min, neutralised by addition of 4M aqueous KOH solution and the solution applied directly to an ion exchange column (SCX), eluting with saturated ammonia in methanol solution to afford the *diamide* **18a** (108 mg, 18%) as an amorphous colourless solid; *R*_t_ (Method: Analysis F) 4.68 min; ν_max_/cm^−1^ (film) 3335 (OH), 3174, 2939 (CH), 2818 (CH), 1632 (NH_2_) and 1602 (C=O); [α]20+4.8 (*c* 1.00 in methanol); δ_H_ (500 MHz; D_2_O) 8.08 (2H, s, 8-H), 7.96 (2H, s, 2-H), 5.85 (2H, d, *J* 4.7, 1′-H), 4.58 (2H, t, *J* 4.7, 2′-H), 4.15 (2H, app. dd, *J* 12.0 and 6.0, 4′-H), 4.09 (2H, t *J* 5.6, 3′-H), 3.08 (4H, s, CH_2_), 2.69-2.65 (4H, m, 5′-H_2_), 2.34-2.27 (4H, m, butyryl 4‑H_2_), 2.16 (6H, s, NMe), 2.04 (4H, t, *J* 7.3, butyryl 2-H_2_) and 1.63-1.54 (4H, m, butyryl 3‑H_2_); δ_C_ (75 MHz; D_2_O) 176.6 (butyryl C-1), 155.7 (C-6), 153.0 (C-2), 148.9 (C-4), 140.3 (C-8), 119.0 (C-5), 88.6 (C-1′), 81.9 (C-4′), 73.8 (C-2′), 72.8 (C-3′), 59.4 (C5′), 56.6 (butyryl C-4), 42.1 (NMe), 39.0 (CH_2_), 34.0 (butyryl C-2) and 22.5 (butyryl C-3); *m/z* (ES^+^) 780 (100%, MNa^+^); (Found: MH^+^ 757.3821; C_32_H_48_N_13_O_8_ requires MH^+^ 756.3774).

***N*,*N*′-Di-[5′-deoxy-5′-*N*-methyl-*N*′-(4-butyryl)]-1,3-diaminopropane 18b**

1,3-Diaminopropane (9.0 µL, 0.11 mmol) was added to a solution of 5′-deoxy-5′-*N*-methyl-*N*′-(4-butyric acid)-2′,3′-*O,O*-(1-methylethylidene)adenosine^2^ (131 mg, 0.322 mmol) in DMF (2 mL), stirred 5 min followed by the addition of PyBOP (168 mg, 0.322 mmol) and DIPEA (56.0 µL, 0.322 mmol). The reaction was stirred for 2.5 d and applied directly to an ion exchange column (SCX), eluting with saturated ammonia in methanol solution, followed by flash chromatography, eluting with 10:90 saturated ammonia in methanol solution–CH_2_Cl_2_ to afford the *intermediate diamide* (87 mg, 96%) as a colourless glass; *R*_f_ 0.13 (10:90 saturated ammonia in methanol solution–CH_2_Cl_2_); ν_max_/cm^−1^ (film) 3324 (NH_2_), 3197 (NH_2_), 2972 (CH), 2928 (CH), 1645 (NH_2_) and 1596 (C=O); [α]20–7.6 (*c* 1.00 in methanol); δ_H_ (500 MHz; methanol-*d_4_*) 8.18 (2H, s, 8-H), 8.12 (2H, s, 2-H), 6.07 (2H, app. s, 1′-H), 5.39 (2H, app. s, 2′-H), 4.87 (2H, dd, *J* 6.0 and 3.4, 3′-H), 4.24 (2H, m, 4′-H), 3.05 (4H, t, *J* 6.8, propane 1-H_2_ and 3-H_2_), 2.54 (4H, app. d, *J* 6.4, 5′-H_2_), 2.25 (4H, t, *J* 7.3, butyryl 4-H_2_), 2.11 (6H, s, NMe), 2.02 (4H, t, *J* 7.3, butyryl 2-H), 1.63-1.53 (4H, m, butyryl 3-H_2_), 1.52 (2H, t, *J* 6.8, propane 2-H_2_), 1.48 (6H, s, Me) and 1.27 (6H, s, Me); δ_C_ (75 MHz; methanol-*d_4_*) 175.0 (butyramide C-1), 156.7 (C-6), 153.3 (C-2), 149.6 (C-4), 141.2 (C-8), 119.9 (C-5), 114.8 (*C*Me_2_), 90.9 (C-1′), 85.4 (C-4′), 84.3 (C-2′), 84.0 (C-3′), 59.7 (C-5′), 57.6 (butyramide C-4), 42.0 (NMe), 37.1 (propyl C-1 and C-3), 34.0 (butyramide C-2), 29.5 (propyl C-2), 26.7 (Me), 24.8 (Me) and 23.2 (butyramide C-3); *m/z* (ES^+^) 851.5 (15%, MH^+^) and 426.2 (100%, MH_2_^2+^); (Found: MH^+^ 851.4622; C_39_H_58_N_14_O_8_ requires MH^+^ 851.4635).

The *intermediate diamide* (52 mg, 0.061 mmol) was treated with 5M aqueous HCl solution (2 mL) for 5 min, neutralised by addition of 4M aqueous NaOH solution and the solution applied directly to an ion exchange column (SCX), eluting with saturated ammonia in methanol solution to afford the *diamide* **18b** (35 mg, 74%) as a colourless glass; *R*_t_ (Method: Analysis F) 4.71 min; ν_max_/cm^−1^ (film) 3318 (OH), 3263 (NH_2_), 3175 (NH_2_), 2939 (CH), 2862 (CH), 1646 (NH_2_) and 1602 (C=O); [α]20+4.8 (*c* 1.00 in methanol); δ_H_ (500 MHz; methanol‑*d_4_*) 8.07 (2H, s, 8-H), 7.94 (2H, s, 2-H), 5.86 (2H, d, *J* 4.7, 1′-H), 4.67 (2H, t, *J* 4.7, 2′-H), 4.26-4.20 (2H, m, 4′-H), 4.14 (2H, t, *J* 4.7, 3′-H), 2.99-2.80 (8H, m, 5′-H_2_ and propane 1-H_2_ and 3-H_2_), 2.52 (4H, t, *J* 7.3, butyryl 4-H_2_), 2.32 (6H, s, NMe), 2.09-2.03 (4H, m, butyryl 2-H_2_), 1.69-1.60 (4H, m, butyryl 3-H_2_) and 1.39 (2H, t, *J* 6.8, propane 2-H_2_); δ_C_ (75 MHz; methanol-*d_4_*) 176.0 (butyramide C-1), 155.6 (C-6), 153.0 (C-2), 148.9 (C-4), 140.6 (C-8), 119.1 (C-5), 88.7 (C-1′), 81.1 (C-4′), 73.5 (C-2′), 72.5 (C-3′), 59.0 (C-5′), 56.7 (butyramide C-4), 41.7 (NMe), 37.0 (propyl C-1 and C-3), 33.8 (butyramide C-2), 28.2 (propyl C-2) and 22.0 (butyramide C-3); *m/z* (ES^+^) 771 (100%, MH^+^) and 386 (100%, MH_2_^2+^); (Found: MH^+^ 771.3998; C_33_H_50_N_14_O_8_ requires MH^+^ 771.4009).

***N*,*N*′-Di-[5′-deoxy-5′-*N*-methyl-*N*′-(4-butyryl)-adenosine]-1,4-diaminobutane 18c**

1,4-Diaminobutane (10.0 µL, 0.0992 mmol) was added to a solution of 5′-deoxy-5′-*N*-methyl-*N*′-(4-butyric acid)-2′,3′-*O,O*-(1-methylethylidene)adenosine^2^ (121 mg, 0.298 mmol) in DMF (2 mL), stirred 5 min followed by the addition of PyBOP (155 mg, 0.298 mmol) and DIPEA (52.0 µL, 0.298 mmol). The reaction was stirred for 2.5 d and the solution applied directly to an ion exchange column (SCX), eluting with saturated ammonia in methanol solution, followed by flash chromatography, eluting with 10:90 saturated ammonia in methanol solution–CH_2_Cl_2_ to afford the *intermediate diamide* (85 mg, 99%) as a colourless glass; *R*_f_ 0.26 (10:90 saturated ammonia in methanol solution–CH_2_Cl_2_); ν_max_/cm^−1^ (film) 3307 (NH_2_), 3181 (NH_2_), 2936 (CH), 2846 (CH), 2802, 1644 (NH_2_) and 1596 (C=O); [α]20–5.2 (*c* 1.00 in methanol); δ_H_ (500 MHz; methanol-*d_4_*) 8.18 (2H, s, 8-H), 8.13 (2H, s, 2-H), 6.07 (2H, d, *J* 2.1, 1′-H), 5.39 (2H, dd, *J* 6.4 and 2.1, 2′-H), 4.87 (2H, dd, *J* 6.4 and 3.0, 3′-H), 4.25 (2H, ddd, *J* 13.3, 7.3 and 3.0, 4′-H), 3.03 (4H, app. t, *J* 5.1, butane 1-H_2_ and 4-H_2_), 2.54 (4H, app. dd, *J* 5.6 and 2.3, 5′-H_2_), 2.25 (4H, t, *J* 7.3, butyryl 4-H_2_), 2.11 (6H, s, NMe), 2.01 (4H, t, *J* 7.3, butyryl 2-H_2_), 1.57 (4H, tt, *J* 7.3 and 7.3, butyryl 3-H_2_), 1.48 (6H, s, Me), 1.39-1.34 (4H, m, butane 2-H_2_ and 3-H_2_) and 1.27 (6H, s, Me); δ_C_ (75 MHz; methanol-*d_4_*) 175.9 (butyramide C-1), 157.7 (C-6), 154.3 (C-2), 150.6 (C-4), 142.3 (C-8), 121.0 (C-5), 115.8 (*C*Me_2_), 91.9 (C-1′), 86.4 (C-4′), 85.3 (C-2′), 85.0 (C-3′), 60.8 (C-5′), 58.6 (butyramide C-4), 43.1 (NMe), 40.3 (butyl C-1 and C-4), 35.1 (butyramide C-2), 28.2 (butyramide C-3), 27.8 (Me), 24.3 (Me) and 24.1 (butyl C-2 and C-3); *m/z* (ES^+^) 865.6 (30%, MH^+^) and 433.4 (100%, MH_2_^2+^); (Found: MH^+^ 865.4813; C_40_H_60_N_14_O_8_ requires MH^+^ 865.4791).

The *intermediate diamide* (30 mg, 0.035 mmol) was treated with 5M aqueous HCl solution (1 mL) for 10 min, neutralised by addition of 4M aqueous NaOH solution and the solution applied directly to an ion exchange column (SCX), eluting with saturated ammonia in methanol solution to afford the *diamide* **18c** (20 mg, 73%) as a colourless glass; *R*_t_ (Method: Analysis G) 4.64 min; ν_max_/cm^−1^ (film) 3291 (NH_2_), 3190 (NH_2_), 2939 (CH), 2868 (CH), 2802, 1632 (NH_2_), 1596 (C=O) and 1569; [α]20–14.8 (*c* 1.00 in water); δ_H_ (500 MHz; D_2_O) 8.10 (2H, s, 8-H), 7.99 (2H, s, 2-H), 5.88 (2H, d, *J* 4.3, 1′-H), 4.61 (2H, app. t, *J* 4.7, 2′‑H), 4.17 (2H, dd, *J* 11.1 and 5.7, 4′-H), 4.12 (2H, app. t, *J* 5.7, 3′-H), 2.90 (4H, m, butane 1-H_2_ and 4-H_2_), 2.74 (4H, m, 5′-H_2_), 2.40-2.30 (4H, m, butyryl 4-H_2_), 2.20 (6H, s, Me), 2.04 (4H, t, *J* 7.3, butyryl 2-H_2_), 1.63 (2H, t, *J* 7.3, butyryl 3-H_A_), 1.60 (2H, t, *J* 7.3, butyryl 3-H_B_) and 1.23 (4H, m, propane 2-H_2_ and 3-H_2_); δ_C_ (75 MHz; D_2_O) 176.15 (butyryl C-1), 155.7 (C‑6), 153.0 (C-2), 148.9 (C-4), 140.4 (C-8), 119.0 (C-5), 88.6 (C-1′), 81.6 (C-4′), 73.6 (C-2′), 72.6 (C-3′), 59.2 (C-5′), 56.5 (butyryl C-4), 42.0 (NMe), 39.2 (butane C-1 and C-4), 34.0 (butyryl C-2), 26.1 (butane C-2 and C-3) and 22.5 (butyryl C-3); *m/z* (ES^+^) 785.5 (100%, MH^+^), 808.4 (MNa^+^) and 393.3 (MH_2_^2+^); (Found: MH^+^ 785.4158; C_34_H_52_N_14_O_8_ requires MH^+^ 785.4165).

***N*,*N*′-Di-[5′-deoxy-5′-*N*-methyl-*N*′-(4-butyryl)-adenosine]-1,6-diaminohexane 18d**

PyBOP (245 mg, 0.471 mmol) and DIPEA (82.0 µL, 0.471 mmol) were added to a solution of 5′-deoxy-5′-*N*-methyl-*N*′-(4-butyric acid)-2′,3′-*O,O*-(1-methylethylidene)adenosine^2^ (160 mg, 0.394 mmol) and 1,6-diaminohexane (18.3 mg, 0.157 mmol) in DMF (2 mL). The reaction was stirred for 20 min and the solution applied directly to an ion exchange column (SCX), eluting with saturated ammonia in methanol solution, followed by flash chromatography (gradient elution: 8:92-10:90 saturated ammonia in methanol solution­–CH_2_Cl_2_ to afford the *intermediate diamide* (134 mg, 96%) as a colourless foam; *R*_f_ 0.21 (10:90 saturated ammonia in methanol solution–CH_2_Cl_2_); ν_max_/cm^−1^ (film) 3330 (NH_2_), 2972 (CH), 2858 (CH), 2803, 2481, 2229, 2134, 2067, 1650 (C=O) and 1599 (NH_2_); [α]20−1.8 (*c* 4.00 in methanol); δ_H_ (500 MHz; methanol-*d_4_*) 8.18 (2H, s, 8-H), 8.13 (2H, s, 2-H), 6.07 (2H, d, *J* 2.1, 1′-H), 5.38 (2H, dd, *J* 6.4 and 2.1, 2′-H), 4.87 (2H, dd, *J* 6.4 and 3.4, 3′-H), 4.26-4.21 (2H, m, 4′-H), 3.01 (4H, t, *J* 7.3, hexyl 1-H_2_ and 6-H_2_), 2.58-2.49 (4H, m, 5′-H_2_), 2.25 (4H, t, *J* 7.3, butyramide 4-H_2_), 2.10 (6H, s, NMe), 2.02 (4H, t, *J* 7.3, butyramide 2-H_2_), 1.63-1.52 (4H, m, butyramide 3-H_2_), 1.47 (6H, s, Me), 1.38-1.30 (4H, m, hexyl 2-H_2_ and 5-H_2_), 1.27 (6H, s, Me) and 1.23-1.15 (4H, m, hexyl 3-H_2_ and 4-H_2_); δ_C_ (75 MHz; methanol-*d_4_*) 176.0 (butyramide C-1), 157.8 (C-6), 154.4 (C-2), 150.7 (C-4), 142.4 (C-8), 121.1 (C-5), 116.0 (*CMe*_2_), 92.0 (C‑1′), 86.5 (C-4′), 85.4 (C-2′), 85.1 (C-3′), 60.9 (C-5′), 58.7 (butyramide C-4), 43.2 (NMe), 40.7 (hexyl C-1 and C-6), 35.2 (butyramide C-2), 30.8 (hexyl C-2 and C-5), 28.0 (hexyl C-3 and C-4), 27.9 (Me), 26.0 (Me) and 24.5 (butyramide C-3); *m/z* (ES^+^) 915.6 (100% MNa^+^), 893.6 (MH^+^) and 447.4 (MH_2_^2+^); (Found: MH^+^  893.5130; C_42_H_64_N_14_O_8_ requires MH^+^ 893.5104).

The *intermediate diamide* was treated with 5M aqueous HCl solution (2 mL) for 10 min, neutralised by addition of 4M aqueous NaOH solution and the solution applied directly to an ion exchange column (SCX) eluting with saturated ammonia in methanol solution to afford the *diamide* **18d** (87 mg, 71%) as a colourless glass; *R*_t_ (Method: Analysis G) 4.84 min; ν_max_/cm^−1^ (film) 3325 (NH_2_), 3269 (NH_2_), 3192 (OH), 2935 (CH), 2851 (CH), 2802, 1647 (NH_2_) and 1603 (C=O); [α]20+3.0 (*c* 4.00 in methanol); δ_H_ (500 MHz; methanol-*d_4_*) 8.17 (2H, s, 8-H), 8.10 (2H, s, 2-H), 5.89 (2H, d, *J* 4.3, 1′-H), 4.62 (2H, app. t, *J* 5.1, 2′-H), 4.15 (2H, app. t, *J* 5.6, 3′-H), 4.09 (2H, app. dd, *J* 11.1 and 5.6, 4′-H), 2.98 (4H, t, *J* 7.3, hexyl 1-H_2_ and 6-H_2_), 2.72-2.68 (4H, m, 5′-H_2_), 2.36 (4H, t, *J* 7.7, butyramide 4-H_2_), 2.20 (6H, s, NMe), 2.06 (4H, t, *J* 7.7, butyramide 2-H_2_), 1.71-1.61 (4H, m, butyramide 3-H_2_), 1.35-1.26 (4H, m, hexyl 2-H_2_ and 5-H_2_) and 1.18-1.12 (4H, m, hexyl 3-H_2_ and 4-H_2_); δ_C_ (75 MHz; methanol-*d_4_*) 175.5 (butyramide C-1), 157.3 (C-6), 153.9 (C-2), 150.5 (C-4), 141.5 (C-8), 120.6 (C-5), 90.6 (C-1′), 83.3 (C-4′), 74.7 (C-2′), 73.7 (C-3′), 60.7 (C-5′), 58.3 (butyramide C-4), 43.0 (NMe), 40.2 (hexyl C-1 and C-6), 34.8 (butyramide C-2), 30.2 (hexyl C-2 and C-5), 27.5 (hexyl C-3 and C-4) and 24.0 (butyramide C-3); *m/z* (ES^+^) 407.2 (100% MH_2_^2+^) and 813.5 MH^+^; (Found: MH^+^ 813.4511; C_36_H_56_N_14_O_8_ requires MH^+^ 813.4478.

***N*,*N*′-Dimethyl-*N*,*N*′-di-[5′-deoxy-5′-*N*-methyl-*N*′-(4-butyryl)- adenosine]-1,3-diaminopropane diiodide 19b**

Methyl iodide (8.8 µL, 0.14 mmol) was added to a solution of the *diamide* **18b** (55 mg, 0.072 mmol) in water (0.8 mL) and acetonitrile (0.5 mL), stirred for 16 h and the solvent removed *in vacuo* to afford the *diiodide* **19b** (59 mg, 78%) as a colourless glass; *R*_t_ (Method: Analysis G) 4.51 min; ν_max_/cm^−1^ 3289 (NH_2_ and OH), 2934 (CH), 1628 (NH_2_) and 1593 (C=O); [α]20+6.3 (*c* 2.15 in D_2_O); δ_H_ (500 MHz; D_2_O) 8.14 (2H, m, 8-H), 8.01 (2H, m, 2‑H), 5.98 (2H, d, *J* 3.4 1′-H), 4.67 (2H, dd, *J* 5.6 and 3.4, 2′-H), 4.50 (2H, app. t, *J* 7.7, 4′-H), 4.38 (2H, app. t, *J* 6.0, 3′-H), 3.94 (2H, dd, *J* 14.5 and 9.8. 5′-H_A_), 3.69 (2H, app. d, *J* 14.5, 5′‑H_B_), 3.38-3.28 (4H, m, butyryl 4-H_2_), 3.15 (6H, s, NMe), 3.11 (6H, s, NMe), 2.86 (4H, t, *J* 6.8, propyl 1-H_2_ and 3-H_2_), 2.15-2.06 (2H, m, butyryl 2-H_A_), 2.01-1.77 (6H, m, butyryl 2-H_B_ and butyryl 3-H_2_) and 1.38 (2H, tt, *J* 6.8, propyl 2-H_2_); δ_C_ (75 MHz; D_2_O) 173.8 (butyryl C-1), 155.5 (C-6), 152.9 (C-2), 148.6 (C-4), 140.4 (C-8), 118.9 (C-5), 89.6 (C-1′), 77.1 (C-4′), 72.6 (C-2′), 72.1 (C-3′), 65.8 (C-5′), 64.2 (butyryl C-4), 52.5 (Me), 52.0 (Me), 36.9 (propane C-1 and C-3), 31.7 (butyryl C-2), 28.0 (propane C-2) and 18.8 (butyryl C-3); *m/z* (ES^+^) 400 (100%, [M-2I^-^]^2+^); (Found: [M-2I^-^]^2+^ 400.2177; C_35_H_56_N_14_O_8_ requires [M-2I^-^]^2+^ 400.2197).

***N*,*N*′-Dimethyl-*N*,*N*′-di-[5′-deoxy-5′-*N*-methyl-*N*′-(4-butyryl)-adenosine]-1,6-diaminohexane diiodide 19d**

Methyl iodide (6.30 µL, 0.101 mmol) was added to a solution of the diamide **18d** (41.0 mg, 0.051 mmol) in acetonitrile (0.4 mL) and water (0.2 mL). The reaction was stirred for 1 d, methyl iodide (3.15 µL, 0.501 mmol) added, stirred for 3 d, methyl iodide (1.56 µL, 0.251 mmol) added, stirred for 7 d and concentrated *in vacuo* to afford the *quaternary ammonium salt* **19d** (44 mg, 79%) as a colourless glass; *R*_t_ (Method: Analysis G) 4.79 min; ν_max_/cm^-1^ (film) 3320 (NH_2_), 3197 (NH_2_), 2934 (CH), 2862, 1645 (NH_2_) and 1602 (C=O); [α]20+1.6 (*c* 2.00 in 1:1 water­–methanol); δ_H_ (500 MHz; methanol-*d_4_*) 8.16 (2H, s, 8-H), 8.06 (2H, s, 2-H), 5.99 (2H, d, *J* 3.0, 1′-H), 4.67-4.63 (2H, m, 2′-H), 4.50 (2H, app. t, *J* 7.7, 3′-H), 4.37 (2H, app. t, *J* 6.0, 4′-H), 3.95 (2H, dd, *J* 14.5 and 4.3, 5′-H_A_), 3.70 (2H, d, *J* 14.5, 5′-H_B_), 3.38-3.26 (4H, m, butyryl 4-H_2_), 3.15 (6H, s, NMe), 3.11 (6H, s, NMe), 2.83 (4H, t, *J* 6.8, hexyl 1-H_2_ and 6-H_2_), 2.12 (2H, tt, *J* 7.3 and 7.3, butyryl 2-H_A_), 2.03-1.79 (6H, m, butyryl 2‑H_B_ and 3-H_2_), 1.21-1.13 (4H, m, hexyl 2-H_2_ and 5-H_2_) and 1.03-0.96 (4H, m, hexyl 3-H_2_ and 4‑H_2_); δ_C_ (75 MHz; methanol-*d_4_*) 173.9 (butyryl C-1), 155.8 (C-6), 153.2 (C-2), 148.9 (C‑4), 140.7 (C-8), 120.3 (C-5), 89.8 (C-1′), 77.2 (C-4′), 72.8 (C-2′), 72.2 (C-3′), 66.0 (C-5′), 64.4 (butyryl C-4), 52.7 (NMe), 52.2 (NMe), 39.6 (hexyl C-1 and C-8), 32.0 (butyryl C-2), 28.5 (hexyl C-2 and C-5), 26.0 (hexyl C-3 and C-4) and 19.0 (butyryl C-3); *m/z* (ES^+^) 421.2 (100% [M−2I^−^]^2+^) and 969.4 [M−I^−^]^+^; (Found: [M−2I^−^]^+^) 841.4770; C_38_H_62_N_14_O_8_ requires [M−2I^−^]^+^ 841.4791).

**2,2′-Bis(azidomethyl)biphenyl S3**

A solution of phosphorus tribromide (92 µL, 0.98 mmol) in CHCl_3_ (1 mL) was added dropwise to a solution of 2,2′-biphenyldimethanol (**S2**) (200 mg, 0.933 mmol) in CHCl_3_ (10 mL), and the reaction mixture was stirred at room temperature for 5 h. The reaction mixture was diluted with CH_2_Cl_2_ (50 mL), washed with water (2 × 50 mL) and brine (50 mL), dried (MgSO_4_) and concentrated *in vacuo* to give a crude product which was purified by flash chromatography, eluting with petrol, to give 2,2′-bis(bromomethyl)biphenyl^3^ (241 mg, 76%) as colourless prisms; m.p. 89-90 °C (lit.^3^ 87-88 °C from acetone); *R*_f_ 0.46 (10:90 EtOAc–CH_2_Cl_2_); ν_max_/cm^−1^ (solid) 3059, 2846, 1474 and 1435; δ_H_ (300 MHz; CDCl_3_) 7.55 (2H, dd, *J* 7.3 and 1.8, 6-H and 6′-H), 7.42 (2H, ddd, *J* 7.3, 7.3 and 1.8, 4-H and 4′-H), 7.37 (2H, ddd, *J* 7.3, 7.3 and 1.5, 5-H and 5′-H), 7.27 (2H, dd, *J* 7.3 and 1.5, 3-H and 3′-H), 4.35 (2H, d, *J* 10.0, 2‑C*H_A_*H_B_ and 2′-C*H_A_*H_B_) and 4.19 (2H, d, *J* 10.0, 2‑CH_A_*H_B_* and 2′-CH_A_*H_B_*); *m/z* (EI^+^) 339.9 (30%, [C_14_H_12_^79^Br^81^Br]^+^).

Sodium azide (45 mg, 0.69 mmol) was added to a stirred solution of 2,2′‑bis(bromomethyl)biphenyl (94 mg, 0.28 mmol) in DMSO (2 mL), and the reaction mixture was stirred at room temperature for 18 h. Water (20 mL) was added, and the mixture was extracted with ether (3 × 50 mL). The combined organics were washed with water (3 × 50 mL) and brine (50 mL), dried (MgSO_4_) and concentrated *in vacuo* to give a crude product which was purified by flash chromatography, eluting with 3:97 EtOAc–petrol, to yield the diazide^4^ **S3** (69 mg, 95%) as a colourless oil; *R*_f_ 0.11 (5:95 EtOAc–petrol); ν_max_/cm^−1^ (film) 3063, 2931, 2096, 1477 and 1447; δ_H_ (300 MHz; CDCl_3_) 7.47 (2H, dd, *J* 7.3 and 1.7, 6-H and 6′-H), 7.44 (2H, ddd, *J* 7.3, 7.3 and 1.3, 5‑H and 5′-H), 7.40 (2H, ddd, *J* 7.3, 7.3 and 1.7, 4-H and 4′-H), 7.22 (2H, dd, *J* 7.3 and 1.3, 3-H and 3′-H), 4.13 (2H, d, *J* 13.7, 2-C*H*_A_CH_B_ and 2′‑C*H*_A_CH_B_) and 4.07 (2H, d, *J* 13.7, 2-CH_A_C*H*_B_ and 2′‑CH_A_C*H*_B_); *m/z* (ES^+^) 287.1 (30%, MNa^+^).

**Biphenyl-2,2′-diyldimethanamine S4**

A solution of the diazide **S3** (63 mg, 0.24 mmol) in THF (15 mL) was added *via* cannula to a solution of triphenylphosphine (250 mg, 0.954 mmol) in THF (10 mL), and the reaction mixture was stirred at room temperature for 96 h. Water (0.5 mL) was added and the reaction mixture was heated at reflux for 2 h. The reaction mixture was cooled to room temperature and concentrated *in vacuo* to give a crude product which was purified by flash chromatography (gradient elution: 2:98-5:95 saturated ammonia in methanol solution–CH_2_Cl_2_) to give the diamine^5^ **S4** (47 mg, 92%) as a pale yellow oil; *R*_f_ 0.11 (5:95 saturated ammonia in methanol solution–CH_2_Cl_2_); ν_max_/cm^−1^ (film) 3352, 3058, 2926, 1573 and 1475; δ_H_ (300 MHz; CDCl_3_) 7.46 (2H, dd, *J* 7.7 and 1.4, 3‑H and 3′-H), 7.38 (2H, ddd, *J* 7.4, 7.4 and 1.5, 4-H and 4′-H), 7.29 (2H, ddd, *J* 7.4, 7.4 and 1.4, 5-H and 5′-H), 7.14 (2H, dd, *J* 7.4 and 1.5, 6-H and 6′‑H), 3.58 (4H, s, 2-CH_2_ and 2′-CH_2_) and 1.62 (4H, br s, NH_2_); *m/z* (ES^+^) 213.2 (100%, MH^+^).

***N*,*N*′-(Biphenyl-2,2′-diylybismethylene)bis{(2′′*S*)-2′′-*tert*-butoxycarbonylamino-4′′-[5′′′-deoxy-5′′′-(methylamino)-2′′′,3′′′-*O*-(1′′′′-ethylethylidene)adenosyl]butanamide} S5**

PyBOP (167 mg, 0.321 mmol) and DIPEA (56 µL, 0.32 mmol) were added to a stirred solution of the diamine **S4** (17 mg, 0.081 mmol) and the acid **6**^2^ (167 mg, 0.321 mmol) in DMF (4 mL) and the reaction mixture was stirred at room temperature for 42 h. The crude product was purified by ion exchange chromatography (SCX), eluting with saturated ammonia in methanol solution, followed by flash chromatography (gradient elution: 6:94-9:91 methanol–CH_2_Cl_2_) to yield a mixture of the diamide and an unwanted side product. A second flash chromatography (gradient elution: 7:93-8:92 methanol–CH_2_Cl_2_) gave the *diamide* **S5** (61 mg, 62%; complex mixture of diastereoisomers) as a colourless foam; *R*_f_ 0.29 (10:90 saturated ammonia in methanol solution–CH_2_Cl_2_); ν_max_/cm^−1^ (film) 3323, 3206, 2979, 2459, 1650, 1599 and 1476; [α]20+16.4 (*c* 1.00 in CH_2_Cl_2_); δ_H_ (500 MHz; methanol-*d*_4_) 8.28‑8.26 (2H, m, Ade 8‑H), 8.24‑8.22 (2H, m, Ade 2-H), 7.46-7.41 (2H, m, 6-H and 6′-H), 7.36‑7.28 (4H, m, 4‑H, 4′-H, 5-H and 5′-H), 7.16‑7.13 (2H, d, *J* 6.4, 3-H and 3′-H), 6.18-6.16 (2H, m, 1′′′‑H), 5.49-5.44 (2H, m, 2′′′‑H), 5.02-4.97 (2H, m, 3′′′-H), 4.46‑4.32 (2H, m, 4′′′-H), 4.24-3.99 (6H, m, 2′′-H, 2-CH_2_ and 2′‑CH_2_), 2.71-2.65 (2H, m, 5′′′‑H_A_), 2.65‑2.59 (2H, m, 5′′′-H_B_), 2.48-2.38 (4H, m, 4′′-H), 2.21 (6H, s, NMe), 1.91‑1.81 (2H, m, 3′′‑H_A_), 1.73-1.64 (2H, m, 3′′‑H_B_), 1.59 (6H, s, CMe_2_), 1.44 (18H, s, *t*-Bu^min^), 1.42 (18H, s, *t*‑Bu^maj^) and 1.38 (6H, s, CMe_2_); δ_C_ (75 MHz; methanol-*d*_4_) 175.0 (amide C=O), 158.0 (carbamate C=O), 157.7, 154.4 (Ade C‑2), 150.6, 142.2 (Ade C-8), 141.0, 137.7, 131.1 (C-3 and C-3′), 129.4 (C-4 and C-4′ or C-5 and C-5′), 129.1 (C-6 and C-6′), 128.4 (C-4 and C-4′ or C-5 and C-5′), 121.0, 115.9 (*C*Me_2_), 91.9 (C‑1′′′^min^), 91.7 (C‑1′′′^maj^), 86.3 (C‑4′′′), 85.3 (C-2′′′), 84.9 (C-3′′′), 81.0 (*C*Me_3_), 61.1 (C‑5′′′^min^), 60.9 (C-5′′′^maj^), 55.9 (C-4′′), 55.1 (C-2′′), 43.3 (NMe^maj^), 43.2 (NMe^min^), 42.3 (2‑CH_2_ and 2′‑CH_2_), 30.6 (C‑3′′), 29.1 (C*Me*_3_), 27.8 (C*Me*_2_) and 26.0 (C*Me*_2_); *m/z* (ES^+^) 1219.6 (100%, MH^+^), 610.3 (50%, MH_2_^2+^); (Found: MH^+^ 1219.6364; C_60_H_82_N_16_O_12_ requires MH^+^ 1219.6371). Analysis of the product by 500 MHz ^1^H NMR spectroscopy revealed that partial epimerisation occurred at the 2′′-position under these conditions.

***N*,*N*′-(Biphenyl-2,2′-diylybismethylene)bis{(2′′*S*)-2′′-amino-4′′-[5′′′-deoxy-5′′′-(methylamino)-adenosyl]butanamide} 9c**

The diamide **S5** (59 mg, 0.048 mmol; complex mixture of diastereoisomers) was dissolved in 5M aqueous HCl solution (1 mL), and the reaction mixture was stirred at room temperature for 10 min. The reaction mixture was neutralised by the dropwise addition of 2M aqueous NaOH solution and the crude product was purified by ion exchange chromatography (SCX), eluting with saturated ammonia in methanol solution, to give the *diamide* **9c** (44 mg, 98%; complex mixture of diastereoisomers) as a colourless foam; ν_max_/cm^−1^ (solid) 3321, 3208, 1642, 1596 and 1418; [α]20–35.6 (*c* 1.00 in DMSO); δ_H_ (500 MHz; DMSO‑*d*_6_) 8.56-8.38 (2H, m, amide NH), 8.35 (2H, s, Ade 8-H^min^), 8.34 (2H, s, Ade 8-H^maj^), 8.17 (2H, s, Ade 2‑H^maj^), 8.16 (2H, s, Ade 2‑H^min^), 7.41‑7.23 (10H, m, Ph and Ade NH_2_), 7.18‑7.12 (2H, m, Ph), 5.88 (2H, d, *J* 5.1, 1′′′-H), 4.69‑4.63 (2H, m, 2′′′-H), 4.29‑4.24 (4H, m, 2-CH_2_^min^ and 2′‑CH_2_^min^), 4.15-4.10 (2H, m, 3′′′‑H), 4.06‑3.98 (6H, m, 4′′′-H, 2-CH_2_^maj^ and 2′-CH_2_^maj^), 3.43‑3.39 (2H, m, 2′′-H), 2.79-2.74 (2H, m, 5′′′-H_A_^min^), 2.72-2.68 (2H, m, 5′′′‑H_A_^maj^), 2.61‑2.55 (2H, m, 5′′′-H_B_), 2.54‑2.43 (4H, m, 4′′-H), 2.21 (6H, s, NMe^min^), 2.17 (6H, s, NMe^maj^), 1.97-1.88 (2H, m, 3′′‑H_A_^min^), 1.83‑1.74 (2H, m, 3′′‑H_A_^maj^) and 1.62-1.51 (2H, m, 3′′‑H_B_); δ_C_ (75 MHz; DMSO‑*d*_6_) 175.4 (C=O^min^), 173.4 (C=O^maj^), 156.4, 153.0 (Ade C‑2), 149.7, 140.2 (Ade C-8), 136.9, 136.1, 129.7 (Ph CH), 128.1 (Ph CH), 127.6 (Ph CH), 127.0 (Ph CH), 119.5, 88.0 (C‑1′′′^maj^), 87.9 (C-1′′′^min^), 82.7 (C‑4′′′^min^), 82.4 (C‑4′′′^maj^), 73.0 (C‑2′′′^maj^), 72.9 (C-2′′′^min^), 72.3 (C-3′′′), 59.9 (C-5′′′), 55.1 (C‑4′′^maj^), 54.7 (C-4′′^min^), 53.5 (C‑2′′), 42.9 (NMe^min^), 42.8 (NMe^maj^), 40.5 (2-CH_2_ and 2′-CH_2_) and 31.2 (C-3′′); *m/z* (ES^+^) 939.7 (90%, MH^+^), 470.5 (100%, MH_2_^2+^); (Found: MH^+^ 939.4689; C_44_H_58_N_16_O_8_ requires MH^+^ 939.4696).

**3,3′-Bis(azidomethyl)biphenyl S7**

*N*-Bromosuccinimide (859 mg, 4.83 mmol) and AIBN (50 mg) were added to a solution of 3,3′‑dimethylbiphenyl (**S6**) (400 mg, 2.19 mmol) in CCl_4_ (4 mL), and the reaction mixture was heated at reflux for 18 h. The reaction mixture was cooled to room temperature, diluted with CH_2_Cl_2_ (100 mL), washed with water (75 mL) and brine (75 mL), dried (MgSO_4_) and concentrated *in vacuo* to give a crude product which was purified by flash chromatography (gradient elution: 0:100-2:98 EtOAc–petrol) to give 3,3′-bis(bromomethyl)biphenyl^6^ (561 mg, 76%) as colourless plates; m.p. 102-104 °C (lit.^6^ 103‑104 °C from benzene); *R*_f_ 0.36 (20:80 EtOAc–petrol); ν_max_/cm^−1^ (film) 3016, 2967, 2298, 1581 and 1402; δ_H_ (300 MHz; CDCl_3_) 7.60 (2H, d, *J* 1.8, 2-H and 2′-H), 7.51 (2H, ddd, *J* 6.9, 2.1 and 1.8, 6-H and 6′-H), 7.45‑7.39 (4H, m, 4-H, 4′-H, 5-H and 5′-H) and 4.56 (4H, s, 3-CH_2_ and 3′-CH_2_); *m/z* (EI^+^) 339.9 (60%, [C_14_H_12_^79^Br^81^Br]^+^), 337.9 (40%, [C_14_H_12_^79^Br_2_]^+^).

Sodium azide (269 mg, 4.14 mmol) was added to a stirred solution of 3,3′‑bis(bromomethyl)biphenyl (560 mg, 1.66 mmol) in DMSO (10 mL), and the reaction mixture was stirred at room temperature for 20 h. The reaction mixture was diluted with ether (150 mL) and washed with water (100 mL). The aqueous layer was extracted with ether (3 × 50 mL), and the combined organics were washed with water (3 × 50 mL) and brine (100 mL), dried (MgSO_4_) and concentrated *in vacuo* to give a crude product which was purified by flash chromatography, eluting with 3:97 EtOAc–petrol, to yield the *diazide* **S7** (583 mg, 82%) as a colourless oil; *R*_f_ 0.24 (5:95 EtOAc–petrol); ν_max_/cm^−1^ (film) 3036, 2929, 2862, 2096 and 1604; δ_H_ (300 MHz; CDCl_3_) 7.57 (2H, ddd, *J* 7.7, 1.5 and 1.5, 6-H and 6′-H), 7.53 (2H, s, 2-H and 2′-H), 7.47 (2H, dd, *J* 7.7 and 7.5, 5-H and 5′‑H), 7.32 (2H, ddd, 7.5, 1.5 and 1.5, 4-H and 4′‑H) and 4.42 (4H, s, 3-CH_2_ and 3′‑CH_2_); δ_C_ (75 MHz; CDCl_3_) 141.6, 136.1, 129.4 (CH), 127.3 (CH), 127.2 (CH), 127.0 (CH) and 54.8 (3-CH_2_ and 3′-CH_2_); *m/z* (EI^+^) 264.1 (50%, M^+^); (Found: M^+^ 264.1130; C_14_H_12_N_6_ requires M^+^ 264.1123).

**Biphenyl-3,3′-diyldimethanamine S8**

A solution of the diazide **S7** (355 mg, 1.34 mmol) in THF (60 mL) was added *via* cannula to a solution of triphenylphosphine (1.41 g, 5.38 mmol) in THF (80 mL), and the reaction mixture was stirred at room temperature for 72 h. Water (1.5 mL) was added and the reaction mixture was heated at reflux for 2 h. The reaction mixture was cooled to room temperature and concentrated *in vacuo* to give a crude product which was purified by flash chromatography (gradient elution: 5:95-7:93 saturated ammonia in methanol solution–CH_2_Cl_2_) to give the diamine^7^ **S8** (173 mg, 61%) as a pale yellow oil; *R*_f_ 0.05 (5:95 saturated ammonia in methanol solution–CH_2_Cl_2_); ν_max_/cm^−1^ (film) 3353, 3031, 2920, 1580 and 1474; δ_H_ (300 MHz; CDCl_3_) 7.55 (2H, br s, 2-H and 2′-H), 7.49 (2H, ddd, *J* 7.7, 1.5 and 1.5, 4‑H and 4′-H or 6-H and 6′‑H), 7.41 (2H, dd, *J* 7.7 and 7.4, 5-H and 5′-H), 7.30 (2H, ddd, *J* 7.4, 1.5 and 1.5, 4-H and 4′-H or 6-H and 6′-H), 3.95 (4H, s, 3-CH_2_ and 3′-CH_2_) and 1.51 (4H, br s, NH_2_); *m/z* (ES^+^) 254.4 (100%, MH^+^+MeCN), 213.3 (60%, MH^+^).

***N*,*N*′-(Biphenyl-3,3′-diylybismethylene)bis{(2′′*S*)-2′′-*tert*-butoxycarbonylamino-4′′-[5′′′-deoxy-5′′′-(methylamino)-2′′′,3′′′-*O*-(1′′′′-ethylethylidene)adenosyl]butanamide} S9**

PyBOP (200 mg, 0.383 mmol) and DIPEA (67 µL, 0.38 mmol) were added to a stirred solution of the diamine **S8** (20 mg, 0.096 mmol) and the acid **6**^2^ (200 mg, 0.383 mmol) in DMF (4 mL) and the reaction was mixture stirred at room temperature for 20 h. The crude product was purified by ion exchange chromatography (SCX), eluting with saturated ammonia in methanol solution, followed by flash chromatography (gradient elution: 6:94-10:90 methanol–CH_2_Cl_2_) to yield a mixture of the diamide and an unwanted side product. A second flash chromatography (gradient elution: 6:94-10:90 methanol–CH_2_Cl_2_) gave the *diamide* **S9** (84 mg, 72%; complex mixture of diastereoisomers) as a colourless foam; *R*_f_ 0.42 (10:90 saturated ammonia in methanol solution–CH_2_Cl_2_); ν_max_/cm^−1^ (film) 3321, 2975, 2932, 2396, 1647 and 1475; [α]20+13.2 (*c* 1.00 in CH_2_Cl_2_); δ_H_ (500 MHz; methanol-*d*_4_) 8.26 (2H, s, Ade 8‑H^maj^), 8.26 (2H, s, Ade 8-H^min^), 8.23 (2H, s, Ade 2-H), 7.57-7.53 (2H, m, 2-H and 2′-H), 7.49 (2H, d, *J* 6.0, 6-H and 6′‑H), 7.35 (2H, dd, *J* 7.3 and 6.0, 5-H and 5′-H), 7.29‑7.23 (2H, m, 4-H and 4′-H), 6.14 (2H, d, *J* 2.1, 1′′′-H), 5.47-5.42 (2H, m, 2′′′‑H), 5.00-4.98 (2H, m, 3′′′-H^min^), 4.96 (2H, dd, *J* 6.4 and 3.4, 3′′′-H^maj^), 4.54-4.37 (4H, m, 3-CH_2_ and 3′‑CH_2_), 4.36-4.31 (2H, m, 4′′′-H), 4.20‑4.00 (2H, m, 2′′-H), 2.67 (2H, dd, *J* 13.3 and 5.6, 5′′′‑H_A_), 2.60 (2H, dd, *J* 13.3 and 7.7, 5′′′‑H_B_), 2.51-2.40 (4H, m, 4′′-H), 2.20 (6H, s, NMe), 1.97‑1.85 (2H, m, 3′′-H_A_), 1.80‑1.70 (2H, m, 3′′‑H_B_), 1.59 (6H, s, CMe_2_^min^), 1.59 (6H, s, CMe_2_^maj^) and 1.42-1.36 (24H, m, CMe_2_ and *t*‑Bu); δ_C_ (75 MHz; methanol-*d*_4_) 175.2 (amide C=O^min^), 175.1 (amide C=O^maj^), 158.0 (carbamate C=O), 157.7, 154.3 (Ade C-2), 150.6, 142.7, 142.2 (Ade C‑8^min^), 142.2 (Ade C‑8^maj^), 140.8, 130.3 (C-5 and C-5′), 127.9 (C-4 and C‑4′), 127.5 (C-2 and C-2′), 127.2 (C-6 and C-6′), 121.0, 115.9 (*C*Me_2_), 91.9 (C-1′′′^min^), 91.8 (C‑1′′′^maj^), 86.2 (C‑4′′′), 85.3 (C-2′′′), 84.9 (C‑3′′′^maj^), 84.9 (C-3′′′^min^), 80.9 (*C*Me_3_), 61.0 (C‑5′′′^min^), 60.9 (C-5′′′^maj^), 55.9 (C-4′′^maj^), 55.8 (C‑4′′^min^), 55.1 (C-2′′), 44.4 (3-CH_2_ and 3′‑CH_2_), 43.2 (NMe^maj^), 43.0 (NMe^min^), 30.7 (C-3′′), 29.0 (C*Me*_3_), 27.8 (C*Me*_2_) and 26.0 (C*Me*_2_); *m/z* (ES^+^) 1219.6 (20%, MH^+^), 610.3 (100%, MH_2_^2+^); (Found: MH^+^ 1219.6351; C_60_H_82_N_16_O_12_ requires MH^+^ 1219.6371). Analysis of the product by 500 MHz ^1^H NMR spectroscopy revealed that partial epimerisation occurred at the 2′′-position under these conditions.

***N*,*N*′-(Biphenyl-3,3′-diylybismethylene)bis{(2′′*S*)-2′′-amino-4′′-[5′′′-deoxy-5′′′-(methylamino)-adenosyl]butanamide} 9d**

The diamide **S9** (32 mg, 0.26 mmol; complex mixture of diastereoisomers) was dissolved in 5M aqueous HCl solution (2 mL) and the reaction mixture was stirred at room temperature for 15 min. The reaction mixture was neutralised by the dropwise addition of 2M aqueous KOH solution, and the crude product was purified by ion exchange chromatography (SCX), eluting with saturated ammonia in methanol solution, to give the *diamide* **9d** (23 mg, 94%; complex mixture of diastereoisomers) as a colourless film; ν_max_/cm^−1^ (solid) 3332, 3186, 2926, 1644, 1601 and 1475; [α]20–26.4 (*c* 0.50 in DMSO); δ_H_ (500 MHz; DMSO-*d*_6_) 8.61 (2H, br s, NH), 8.35 (2H, s, Ade 8-H^min^), 8.34 (2H, s, Ade 8-H^maj^), 8.17 (2H, s, Ade 2-H), 7.54 (2H, s, 2-H and 2′-H), 7.50 (2H, d, *J* 7.7, 6-H and 6′‑H), 7.40 (2H, ddd, *J* 7.7, 7.3 and 1.7, 5‑H and 5′-H), 7.30 (4H, br s, Ade NH_2_), 7.26 (2H, d, *J* 7.3, 4-H and 4′-H), 7.88 (2H, d, *J* 5.1, 1′′′‑H), 4.65 (2H, dd, *J* 5.1 and 4.7, 2′′′-H), 4.38-4.35 (4H, m, 3-CH_2_ and 3′‑CH_2_), 4.13‑4.09 (2H, m, 3′′′‑H), 4.05‑4.00 (2H, m, 4′′′-H), 3.47-3.43 (2H, m, 2′′‑H), 2.72-2.67 (2H, m, 5′′′-H_A_), 2.60‑2.56 (2H, m, 5′′′-H_B_), 2.49‑2.44 (4H, m, 4′′-H), 2.17 (6H, s, NMe^maj^), 2.16 (6H, s, NMe^min^), 1.88-1.80 (2H, m, 3′′-H_A_) and 1.67-1.59 (2H, m, 3′′-H_B_); δ_C_ (75 MHz; DMSO‑*d*_6_) 173.8 (C=O), 156.2, 153.0 (Ade C-2), 149.6, 140.5, 140.3 (Ade C-8), 129.3 (C-5 and C-5′), 126.7 (C-4 and C-4′), 126.0 (C-2 and C‑2′ or C-6 and C-6′), 125.6 (C-2 and C-2′ or C-6 and C‑6′), 119.4, 88.0 (C-1′′′), 82.4 (C-4′′′^maj^), 82.3 (C-4′′′^min^), 73.0 (C-2′′′), 72.3 (C-3′′′), 59.9 (C‑5′′′^min^), 59.8 (C‑5′′′^maj^), 54.7 (C‑4′′^min^), 54.6 (C‑4′′^maj^), 53.5 (C-2′′^maj^), 53.5 (C‑2′′^min^), 42.6 (NMe), 42.5 (3-CH_2_ and 3′-CH_2_), 31.3 (C‑3′′^maj^), 31.2 (C-3′′^min^) and one signal missing or overlapped; *m/z* (ES^+^) 939.5 (100%, MH^+^); (Found: MH^+^ 939.4664; C_44_H_58_N_16_O_8_ requires MH^+^ 939.4696).

**5′-[{(3′′*S*)-4′′-Propargylamino-3′′-*tert*-butoxycarbonylamino-4′′-oxobutyl}methylamino]-5′-deoxy-2′,3′-*O*-(1′′′-methylethylidene)adenosine S10**

DIPEA (28 µL, 0.16 mmol) was added to a solution of the acid **6**^2^ (84 mg, 0.16 mmol), propargyl amine (9.2 µL, 0.13 mmol) and PyBOP (83 mg, 0.16 mmol) in DMF (2 mL) and the reaction mixture was stirred at room temperature for 18 h. The crude product was purified by ion exchange chromatography (SCX), eluting with saturated ammonia in methanol solution, followed by flash chromatography (gradient elution: 4:96-6:94 saturated ammonia in methanol solution–CH_2_Cl_2_) to yield the *amide* **S10** (63 mg, 70%; 1:1 mixture of diastereoisomers) as a colourless film; *R*_f_ 0.32 (10:90 methanol–CH_2_Cl_2_); ν_max_/cm^−1^ (film) 3318, 3197, 2977, 2796, 2580, 1654, 1599 and 1367; [α]20+4.0 (*c* 1.00 in CH_2_Cl_2_); δ_H_ (500 MHz; methanol-*d*_4_) 8.32 (1H, s, 8-H^maj^), 8.30 (1H, s, 8-H^min^), 8.26 (1H, s, 2-H^min^), 8.26 (1H, s, 2-H^maj^), 6.22-6.19 (1H, m, 1′-H), 5.52-5.48 (1H, m, 2′-H), 5.05-5.01 (1H, m, 3′‑H), 4.41‑4.37 (1H, m, 4′‑H), 4.15-3.88 (3H, m, 3′′-H and propargyl 1-H), 2.75-2.64 (2H, m, 5′-H), 2.62-2.58 (1H, m, propargyl 3-H), 2.54‑2.41 (2H, m, 1′′-H), 2.26 (3H, s, NMe), 1.92‑1.81 (1H, m, 2′′-H_A_), 1.77‑1.68 (1H, m, 2′′‑H_B_), 1.63 (3H, s, CMe_2_) and 1.45-1.41 (12H, m, CMe_2_ and *t*‑Bu); δ_C_ (75 MHz; methanol‑*d*_4_) 174.8 (amide C=O^maj^), 174.7 (amide C=O^min^), 158.0 (carbamate C=O), 157.7, 154.4 (C-2), 150.6, 142.2 (C-8), 121.0, 116.0 (*C*Me_2_), 91.9 (C‑1′^min^), 91.8 (C‑1′^maj^), 86.2 (C-4′^maj^), 86.2 (C‑4′^min^), 85.4 (C-3′^min^), 85.3 (C-3′^maj^), 84.9 (C‑2′^maj^), 84.9 (C‑2′^min^), 80.9 (*C*Me_3_), 80.9 (propargyl C-2), 72.6 (propargyl C-3^min^), 72.6 (propargyl C-3^maj^), 61.1 (C-5′^min^), 60.9 (C-5′^maj^), 55.7 (C-1′′^maj^), 55.6 (C-1′′^min^), 54.9 (C-3′′^min^), 54.7 (C-3′′^maj^), 43.2 (NMe^min^), 43.0 (NMe^maj^), 30.6 (propargyl C-1), 29.8 (C-2′′), 29.0 (C*Me*_3_), 27.8 (C*Me*_2_) and 25.9 (C*Me*_2_); *m/z* (ES^+^) 559.3 (100%, MH^+^); (Found: MH^+^ 559.2995; C_26_H_38_N_8_O_6_ requires MH^+^ 559.2987). Analysis of the product by 500 MHz ^1^H NMR spectroscopy revealed that partial epimerisation occurred at the 3′′-position under these conditions.

***N*,*N*′-(Hexa-2,4-diyne-1,6-diyl)bis{(2′*S*)-2′-*tert*-butoxycarbonylamino-4′-[5′′‑deoxy-5′′-(methylamino)-2′′,3′′-*O*-(1′′′-methylethylidene)adenosyl]butanamide} S11**

CuCl (17 mg, 0.18 mmol) was added to a solution of the amide **S10** (98 mg, 0.18 mmol) in pyridine (3 mL), and the reaction mixture was stirred at room temperature, open to air, for 20 h. The reaction mixture was filtered through celite, washing with CH_2_Cl_2_ (250 mL), and the filtrate was concentrated *in vacuo* and purified by flash chromatography (gradient elution: 8:92-10:90 methanol–CH_2_Cl_2_) to give the *diamide* **S11** (56 mg, 57%; complex mixture of diastereoisomers) as a colourless film; *R*_f_ 0.31 (10:90 methanol–CH_2_Cl_2_); ν_max_/cm^−1^ (film) 3328, 3208, 2979, 2807, 2346, 1648, 1599 and 1367; [α]20+18.0 (*c* 1.00 in CH_2_Cl_2_); δ_H_ (300 MHz; methanol‑*d*_4_) 8.21 (2H, s, Ade 8-H^maj^), 8.19 (2H, s, Ade 8-H^min^), 8.16 (2H, s, Ade 2-H), 6.10 (2H, d, *J* 2.3, 1′′‑H), 5.42-5.37 (2H, m, 2′′-H), 4.94-4.89 (2H, m, 3′′-H), 3.30-3.24 (2H, m, 4′′‑H), 4.04-3.86 (6H, m, 2′-H, 1-H and 6-H), 2.62-2.51 (4H, m, 5′′-H), 2.41‑2.31 (4H, m, 4′‑H), 2.16 (6H, s, NMe^min^), 2.14 (6H, s, NMe^maj^), 1.83-1.70 (2H, m, 3′‑H_A_), 1.65‑1.55 (2H, m, 3′-H_B_), 1.52 (6H, s, CMe_2_), 1.33 (18H, s, *t*-Bu^maj^), 1.32 (18H, s, *t*‑Bu^min^) and 1.30 (6H, s, CMe_2_); δ_C_ (75 MHz; methanol-*d*_4_) 174.8 (amide C=O), 158.0 (carbamate C=O), 157.7, 154.4 (Ade C-2), 150.6, 142.3 (Ade C-8), 120.9, 116.0 (*C*Me_2_), 91.9 (C-1′′^min^), 91.8 (C‑1′′^maj^), 86.2 (C‑4′′), 85.4 (C-2′′), 84.9 (C-3′′^maj^), 84.9 (C‑3′′^min^), 81.0 (*C*Me_3_), 75.9 (C-2 and C‑5), 67.9 (C-3 and C-4), 61.2 (C-5′′^min^), 60.9 (C‑5′′^maj^), 55.8 (C-4′), 55.1 (C‑2′), 43.2 (NMe^maj^), 43.0 (NMe^min^), 30.6 (C-3′), 30.3 (C-1 and C-6), 29.0 (C*Me*_3_), 27.8 (C*Me*_2_) and 26.0 (C*Me*_2_); *m/z* (ES^+^) 1115.6 (15%, MH^+^), 558.3 (100%, MH_2_^2+^); (Found: MH^+^ 1115.5744; C_52_H_74_N_16_O_12_ requires MH^+^ 1115.5745). Analysis of the product by 300 MHz ^1^H NMR spectroscopy revealed that partial epimerisation occurred at the 2′-position under these conditions.

***N*,*N*′-(Hexa-2,4-diyne-1,6-diyl)bis{(2′*S*)-2′-amino-4′-[5′′‑deoxy-5′′-(methylamino)-adenosyl]butanamide} 9e**

The diamide **S11** (24 mg, 0.022 mmol; complex mixture of diastereisomers) was dissolved in 5M aqueous HCl solution (0.5 mL), and the reaction mixture was stirred at room temperature for 15 min. The reaction mixture was neutralised by the dropwise addition of 2M aqueous NaOH solution and the crude product was purified by ion exchange chromatography (SCX), eluting with saturated ammonia in methanol solution, to give the *diamide* **9e** (15 mg, 84%; complex mixture of diastereoisomers) as a colourless foam; ν_max_/cm^−1^ (solid) 3333, 2252, 2126, 1645, 1601 and 1477; [α]20–17.2 (*c* 1.00 in DMSO); δ_H_ (500 MHz; DMSO‑*d*_6_) 8.36 (2H, s, Ade 8-H), 8.17 (2H, s, Ade 2-H^min^), 8.16 (2H, s, Ade 2‑H^maj^), 7.29 (4H, br s, Ade NH_2_), 5.87 (2H, d, *J* 5.4, 1′′-H), 5.46 (2H, br s, 2′-NH_2_), 5.23 (2H, br s, 2′-NH_2_), 4.67‑4.65 (2H, m, 2′′-H), 4.13-4.08 (6H, m, 3′′‑H, 1-H and 6-H), 4.01-3.97 (2H, m, 4′′‑H), 3.53‑3.48 (2H, m, 2′-H), 2.76-2.68 (4H, m, 5′′‑H), 2.58-2.42 (4H, m, 4′-H), 2.19 (6H, s, NMe^maj^), 2.18 (6H, s, NMe^min^), 1.92-1.84 (2H, m, 3′-H_A_) and 1.50-1.43 (2H, m, 3′-H_B_); δ_C_ (75 MHz; DMSO-*d*_6_) 174.7 (C=O), 156.4, 153.0 (Ade C-2), 149.0, 140.2 (Ade C-8), 119.5, 88.6 (C-1′′), 83.1 (C-4′′), 76.1 (C-2 and C-5), 72.9 (C-2′′), 72.3 (C-3′′), 70.2 (C-3 and C-4), 59.9 (C-5′′), 56.1 (C-4′), 43.0 (NMe), 31.0 (C-3′), 25.5 (C-1 and C-6) and one signal missing or overlapped; *m/z* (ES^+^) 835.3 (20%, MH^+^), 418.3 (100%, MH_2_^2+^); (Found: MH_2_^2+^ 418.2069; C_36_H_50_N_16_O_8_ requires MH_2_^2+^ 418.2072).

**Fluorescence anisotropy**

Analysis of novel SAM analogues binding to MetJ in the presence of operator DNA was carried out using fluorescence anisotropy. Fluorescence anisotropy experiments were performed on a Spex Fluorolog Tau spectrofluorometer (HORIBA Jobin Yvon) controlled by Datamax software. The excitation and emission wavelengths were set to 488 nm and 517 nm respectively. Slit widths were set to 5 nm and all experiments were carried out at 19 °C. The detector integration time was 5 seconds and three readings were taken of each sample to obtain an average anisotropy. Free anisotropy values were 0.051-0.060. Increase in fluorescence anisotropy was observed upon titration of MetJ into a solution of F-*metC* (10 nM) with and without ligand (2 μM) in TK buffer pH 7.6 (50 mM Trizma hydrochloride pH 7.6, 100 mM KCl) with a total starting volume of 200 µL. During titrations, the reaction volume did not increase by more than 10%. The final DMSO concentration was 2% or less. After each addition, the sample was mixed with a pipette and left to equilibrate for five minutes before the fluorescence anisotropy readings were acquired. The intensity of linear and perpendicular light, compared to the excitation light is measured experimentally and the anisotropy was calculated using the following equation:‖

$$r=\frac{\text{(F}_{\text{||}}-\text{F}_{\perp}\text{)}}{\text{(F}_{\text{||}}+\text{2F}_{\perp}\text{)}}$$

Where r = fluorescence anisotropy

F_||_ = fluorescence intensity parallel to the excitation plane

F_┴_ = fluorescence intensity perpendicular to the excitation plane

The data was fitted to a sigmoidal growth logistic model using the following equation:

Where A1 refers to the initial value, A2 refers to the final value, x0 refers to the centre and p is a value relating to the slope of the transition. Errors refer the standard deviation of three titration repeats. An arbitrary weighting function with the following equation was employed:

$$\text{w}=\left(\frac{1}{\text{st.dev.of y}}\right)^{2}$$

The fitting was carried out using OriginPro 7.5 software.

F-*metC* was obtained as two complementary single stranded oligonucleotides from MWG:

5′ TAG ACA TCC AGA CGT ATA 3′ (with 5′-fluorescein)

5′ TAT ACG TCT GGA TGT CTA 3′

The fluorescein-labelled anti-Met-box DNA was also obtained as two complementary strands from MWG:

5′ TCC GGC AGG CCG GCA GGA 3′ (with 5′-fluorescein)

5′ TCC TGC CGG CCT GCC GGA 3′

A 2:1 ratio of unlabelled–labelled oligonucleotides were heated to 80 °C in a water-bath. The water-bath was switched off and the mixture was allowed to cool to room temperature.

**Supplementary references**

1.Still, W. C.; Kahn, M., Mitra, A. *J. Org. Chem.* 1978, *43*, 2923.

2.Joce, C.; Caryl, J.; Stockley, P. G.; Warriner, S., Nelson, A. *Org. Biomol. Chem.* 2009, *7*, 635.

3.Tolbert, L. M., Ali, M. Z. *J. Org. Chem.* 1982, *47*, 4793.

4.Alajarin, M.; Bonillo, B.; Sanchez-Andrada, P.; Vidal, A., Bautista, D. *J. Org. Chem.* 2007, *72*, 5863.

5.Hiatt, R.; Shaio, M.-J., Georges, F. *J. Org. Chem.* 1979, *44*, 3265.

6.Wenner, W. *J. Org. Chem.* 1952, *17*, 523.

7.Tobe, Y.; Sasaki, S.; Mizuno, M.; Hirose, K., Naemura, K. *J. Org. Chem.* 1998, *63*, 7481.
